# Integrating multiple ‘omics’ analyses identifies serological protein biomarkers for preeclampsia

**DOI:** 10.1186/1741-7015-11-236

**Published:** 2013-11-06

**Authors:** Linda Y Liu, Ting Yang, Jun Ji, Qiaojun Wen, Alexander A Morgan, Bo Jin, Gongxing Chen, Deirdre J Lyell, David K Stevenson, Xuefeng B Ling, Atul J Butte

**Affiliations:** 1 Department of Pediatrics, Stanford University, 1265 Welch Road, Room X-163 MS-5415, Stanford, CA 94305, USA; 2 Department of Surgery, Stanford University, Stanford, CA 94305, USA; 3 Department of Obstetrics & Gynecology, Stanford University, 269 Campus Drive, CCSR 4245A, Stanford, CA 94305, USA

**Keywords:** 2D gel (two-dimensional gel electrophoresis), LCMS, Multiplex analysis, Preeclampsia, Proteomic profile

## Abstract

**Background:**

Preeclampsia (PE) is a pregnancy-related vascular disorder which is the leading cause of maternal morbidity and mortality. We sought to identify novel serological protein markers to diagnose PE with a multi-’omics’ based discovery approach.

**Methods:**

Seven previous placental expression studies were combined for a multiplex analysis, and in parallel, two-dimensional gel electrophoresis was performed to compare serum proteomes in PE and control subjects. The combined biomarker candidates were validated with available ELISA assays using gestational age-matched PE (n=32) and control (n=32) samples. With the validated biomarkers, a genetic algorithm was then used to construct and optimize biomarker panels in PE assessment.

**Results:**

In addition to the previously identified biomarkers, the angiogenic and antiangiogenic factors (soluble fms-like tyrosine kinase (sFlt-1) and placental growth factor (PIGF)), we found 3 up-regulated and 6 down-regulated biomakers in PE sera. Two optimal biomarker panels were developed for early and late onset PE assessment, respectively.

**Conclusions:**

Both early and late onset PE diagnostic panels, constructed with our PE biomarkers, were superior over sFlt-1/PIGF ratio in PE discrimination. The functional significance of these PE biomarkers and their associated pathways were analyzed which may provide new insights into the pathogenesis of PE.

## Background

As the leading cause of maternal morbidity and mortality, preeclampsia (PE) is a pregnancy-related vascular disorder affecting 5% to 8% of all pregnancies [1,2]. PE, which often associates with fetal growth restriction and pre-term delivery as well as fetal mortality and morbidity, can be remedied by delivery of the placenta and fetus [3]. The etiology of PE is incompletely understood*.* Current diagnosis of PE is based on the signs of hypertension and proteinuria [4], but lacks sensitivity and specificity and carries a poor prognosis for adverse maternal and fetal outcomes [5]. Thus, there is a need to identify PE biomarkers that can provide a definitive diagnosis with the opportunity for better monitoring of the condition’s progression and, thus, improved outcomes and economic benefits.

Although the pathophysiology remains largely elusive, PE is a multisystem disorder of pregnancy with the placenta playing a pivotal role. Investigators have used genetic, genomic and proteomic approaches to compare PE and control placental tissues. Transcriptional profiling of case–control samples has identified disease-specific expression patterns, canonical pathways and gene-gene networks [6-12]. Proteomics-based biomarker studies [13-15] have also revealed candidate biomarkers for future testing. Placental angiogenic and anti-angiogenic factor imbalance, elevated soluble fms-like tyrosine kinase (sFlt-1) and decreased placental growth factor (PIGF) levels are suggested in the pathogenesis of PE [16-22], and the sFlt-1/PIGF ratio has been proposed as a useful index in the diagnosis and management of PE [23,24]. However, no widely applicable, sensitive and specific molecular PE test in routine clinical practice is currently available.

In light of these considerations, there is a strong rationale and need to discover diagnostic biomarkers for PE. It is likely unrealistic that a single biomarker could be used to diagnose multifactorial diseases such as PE. Therefore, we employed a comprehensive unbiased multi-’omics’ approach, integrating results from microarray multiplex analysis and proteomic identification by two-dimensional gel analysis. Our applied parametric method [25,26] allowed us to identify consistent and significant differential gene expression across experiments to develop biomarkers for downstream experimental validation. Serum proteins are routinely used to diagnose diseases, but sensitive and specific biomarkers are hard to find; this may be due to their low serological abundance, which can easily be masked by highly abundant proteins. Our serum protein marker discovery method [27] combines antibody-based serum abundant protein depletion and two-dimensional gel comparative profiling to discover differential protein gel spots between PE and control sera for subsequent protein mass spectrometric identification. We hypothesized that there would be differential serological signatures allowing PE diagnosis. To validate our discovery findings, we tested all the candidates with available ELISA assays, a higher-throughput method. To construct and optimize a sensitive and specific biomarker panel with the least number of protein analytes, a genetic algorithm was used. Close examination of the biomarkers from comparative transcriptomics and proteomics, and their associated pathways led to new hypothesis about their role in PE pathophysiology.

The results validated our hypothesis that sensitive and specific serological biomarker panels can be constructed to diagnose PE. To our knowledge, this represents the first study to employ a muti-’omics’-based biomarker approach to uncover novel PE biomarkers superior to sFlt-1, PIGF, and the sFlt-1/PIGF ratio in PE discrimination. We believe that the functional significance of these PE biomarkers and their associated pathways will provide new insights into the disease pathogenesis and potentially lead to effective novel therapeutics.

## Methods

### Ethics

All the serum samples were purchased from ProMedDX Inc. (Norton, MA, USA, http://www.promeddx.com) and included detailed case report forms. We confirmed with ProMedDX that all of the ProMedDX specimens we used were collected under Institutional Review Board approved protocols by qualified Investigator sites. These sites conducted ProMedDX studies according to 21 CFR, ICH/GCP guidelines and HIPAA Privacy Regulations. Informed consent was obtained from every subject, unless this requirement had been determined by the IRB not to apply and had been waived.

### Study design

**Figure 1:**
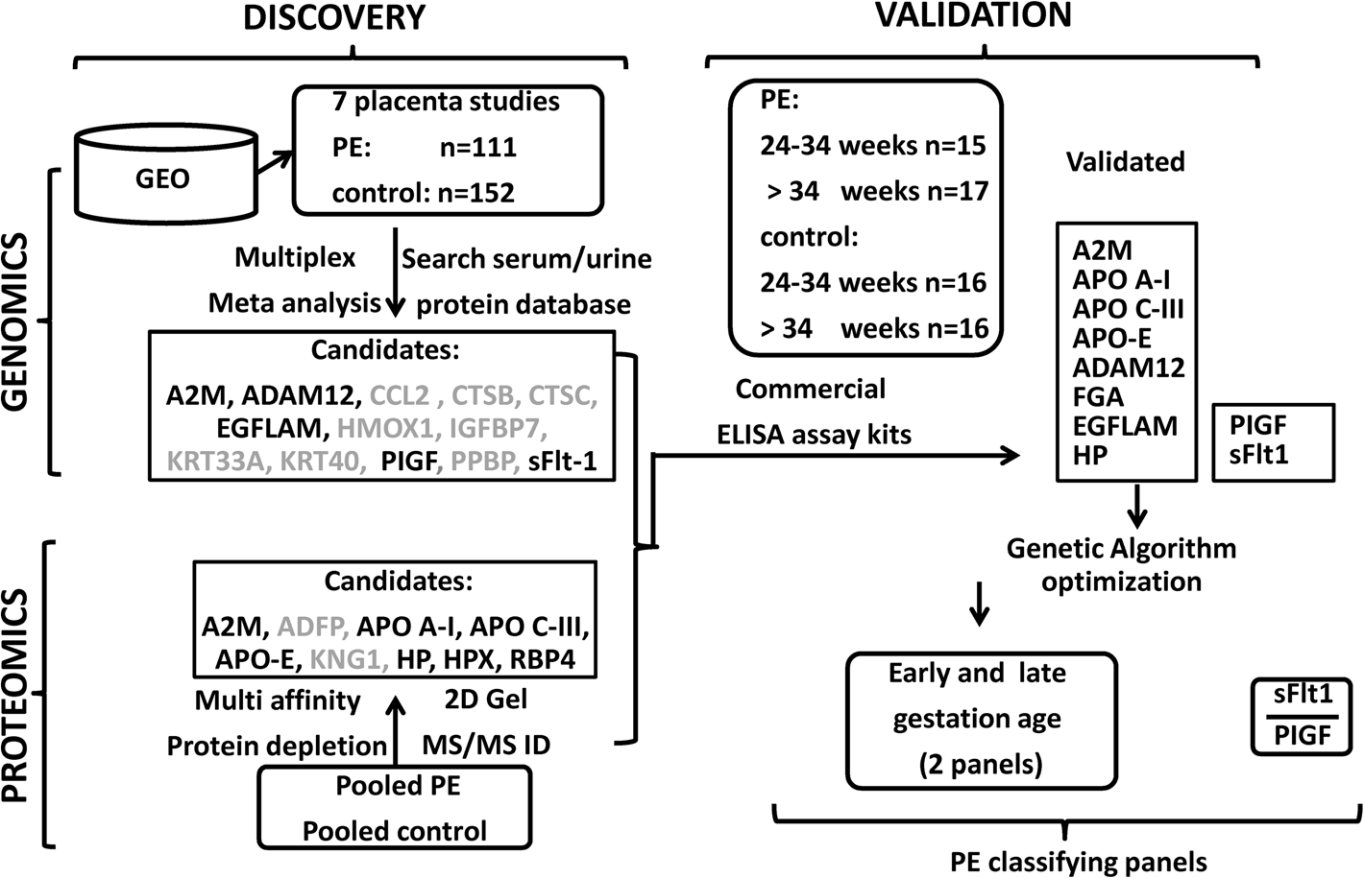
**Study outline of the multi-’omics’-based discovery and validation of PE biomarkers.** Candidate analytes, which failed subsequent validation, are greyed out. PE, preeclampsia.

The overall sample allocation, PE biomarker discovery, validation and diagnostic panel construction steps are illustrated in Figure 1. Our study was conducted in two phases: (1) the discovery phase, which included both the *in silico* expression analysis (n = 111 PE and n = 152 control placenta samples) and the proteomics two-dimensional gel profiling (pooled n = 5 PE and pooled n = 5 control serum proteomes); and (2) the validation phase, which comprised the analysis of independent PE (n = 32) and control (n = 32) cohorts. All the serum samples were purchased from ProMedDX Inc. All serum samples were collected after informed consent was obtained and included detailed case report forms. Excluded from this study were patients who were current smokers, had a history of substance abuse, used *in vitro* fertilization assistance, had chronic hypertension and pregnancies complicated by intrauterine growth restriction. Case (PE) and control (normal pregnant) cohorts were matched for gestational age, ethnicity and parity.

### Multiplex analysis of expression comparing preeclampsia and control placentas

As shown in Additional file 1: Table S1, seven PE placenta expression studies [7,10-12,28,29] were combined and subjected to multiplex analysis with the method we previously developed [25,26]. For each of the 22,394 genes tested, we calculated the meta-fold change across all studies. For gene expression measurements, this corresponds to combining fold-changes across studies to identify a meta-fold-change that is an amalgamation of the constituent studies. We took a linear combination of effect sizes (fold-changes in this case), weighted by the variance in the effect size within each study, with the confidence intervals combined with the same weights. This means that studies with larger intra-study variation (noise) contribute less to the overall estimate of fold-change. The meta *p*-values were obtained by Fisher’s method. Significant genes were selected if they were measured in five or more studies and the meta effect *p* value was less than 4.5 × 10^-5^. We then filtered the gene sets through a list of 3,638 proteins with known detectable abundances in sera, plasma, or urine [30]. The 3,638 protein list was created from public sources [31-34] and has been described [30]. This effort yielded a set of candidate protein biomarkers. At every step of the data mining processes, a biomarker specialist manually curated candidates to provide quality control and prioritize the candidates for the subsequent validation studies.

### Two-dimensional gel analysis comparing pooled preeclampsia and control patient serum samples

To enrich samples for lower abundance serum proteins, serum samples were depleted of the top fourteen serum-abundant proteins (albumin, immunoglobulin G (IgG), antitrypsin, IgA, transferrin, haptoglobin, fibrinogen, alpha2-macroglobulin, alpha1-acid glycoprotein, IgM, apolipoprotein A-I, apolipoprotein A-II, complement C-III and transthyretin) using the Agilent Multiple Affinity Removal System (Agilent, Santa Clara, CA, USA). Specifically, the depletion enabled the increased loading of the remaining proteins by fifteen-fold [27]. Further sample processing, two-dimensional gel electrophoresis, comparative analysis, and differential gel spot protein identification via mass spectrometry was performed as previously described [27].

### ELISA assays validating preeclampsia marker candidates

All assays were ELISA assays and were performed using commercial kits following the vendors’ instructions. All assays were performed to measure serum levels of selected analytes: alpha-2-macroglobin (A2M, Abnova Inc.,Taipei, Taiwan); disintegrin and metalloproteinase domain-containing protein 12 (ADAM12, Mybiosource, San Diego, CA, USA); adipophilin (ADRP, Biotang Inc., Waltham, MA, USA); apolipoprotein (APO) A-I (Abcam Inc., Cambridge, MA, USA); apolipoprotein (APO) C-III (Abnova); apolipoprotein (APO)-E (Abcam); cathepsin B (CTSB, Abcam); cathepsin C (CTSC, USCN Life Science, Wuhan, China); chemokine (C-C motif) ligand 2 (CCL2) (Abnova); haptoglobin (HP, Abcam); hemopexin (HPX, Abcam); PIGF (R&D Systems, Inc., Minneapolis, MN, USA); heme oxygenase 1 (HMOX1, Biotang); insulin-like growth factor binding protein 7 (IGFBP7, USCN Life Science); keratin 33A (KRT33A, USCN Life Science); keratin 40 (KRT40, USCN Life Science); kininogen 1 (KNG1, Abcam); pikachurin (EGFLAM, EIAab Science, Wuhan, China); pro-platelet basic protein (PPBP, Abnova); retinol-binding protein 4 (RBP4, Abcam); and sFlt-1 (R&D Systems).

### Statistical analyses

Patient demographic data were analyzed using the ‘Epidemiological calculator’ (R epicalc package). Student’s *t* test was performed to calculate *p* values for continuous variables, and Fisher’s exact test was used for comparative analysis of categorical variables. Forest plotting with the R rmeta package was used both to represent the placental expression meta analysis and to graphically summarize the serum protein ELISA results. Case (PE) and control samples are not paired; thus, the initial serum protein forest plot analysis should be interpreted with caution. The bootstrapping method was used to create ‘paired’ samples from case and control groups for the subsequent forest plotting analysis of the ELISA results. Therefore, serum protein forest plot analysis provides an overall effect estimation of each analyte’s capability in discriminating PE and normal pregnant control subjects. Hypothesis testing was performed using Student’s *t*-test (two tailed) and the Mann–Whitney *U*-test (two tailed), and local false discovery rate (FDR) [35] to correct for multiple hypothesis testing issues. Biomarker feature selection and panel optimization was performed using a genetic algorithm (R genalg package). The diagnostic performance of each biomarker panel analysis was evaluated by receiver operating characteristics (ROC) curve analysis [36,37]. The biomarker panel score was defined as the ratio between the geometric means of the respective up- and down-regulated protein biomarkers in the maternal circulation.

## Results

### Multi-’omics’-based discovery revealing preeclampsia marker candidates

As shown in Figure 1, previous placental expression studies were combined for a multiplex analysis to discover biomarker candidates that could be used to diagnose PE from normal controls. This effort identified A2M, ADAM12, CCL2, CTSB, CTSC, EGFLAM, HOMX1, IGFBP7, KRT33A, KRT40, PIGF, PPBP and sFlt-1 as differential placental biomarkers for PE. In parallel, two-dimensional gel analysis was performed to compare serological PE and control pooled proteomes, revealing highly discriminating protein spots that were later sequenced. The two-dimensional gel profiling led to the identification of A2M, ADFP, APO A-I, APO C-III, APO-E, KNG1, HP, HPX and RBP4 marker candidates.

### Sample characteristics

The PE and control subjects used for serological protein biomarker validation can be divided into early (PE, n = 15; control, n = 16) and late (PE, n = 17; control, n = 16) gestation groups. As summarized in Tables 1 and 2, no significant differences in age (*p* value, early 0.89, late 0.857, overall 0.6), gestational age (*p* value, early 0.851, late 0.895, overall 0.824) at enrollment, ethnicity (*p* value, early 0.57, late 0.123, overall 0.289), or subjects’ concurrent medical conditions and other clinical features (*p* value, overall 0.35) were observed.

**Table 1:** Ethnicity, age and week of gestation

**Characteristic**	**Early stage**			**Late stage**			**Overall**
	**PE**	**Control**	***p*** **value**	**PE**	**Control**	***p*** **value**	***p*** **value**
	**Number = 15 (48.4%)**	**Number = 16 (51.6%)**		**Number = 17 (51.5%)**	**Number = 16 (48.5%)**		
Ethnicity			0.57			0.123	0.289
African American	5 (33.3%)	5 (31.2%)		2 (11.8%)	4 (25%)		
Asian	2 (13.3%)	0 (0)		0 (0)	0 (0)		
Hispanic	8 (53.3%)	10 (62.5%)		11 (64.7%)	12 (75%)		
Other	0 (0)	1 (6.2%)		4 (23.5%)	0 (0)		
Age (year)							
mean (SD)	24.3 (4.5)	24.1 (6.1)	0.89	27.9 (9.0)	26.6 (7.7)	0.857	0.6
Week of gestation^a^							
mean (SD)	30.3 (3.2)	30.1 (2.9)	0.851	37.1 (1.4)	37.2 (1.6)	0.895	0.824

^a^The time of blood drawing. PE, preeclampsia.

**Table 2:** Concurrent medical conditions and clinical features

**Characteristic**	**PE**	**Control**	***p*** **value**
	**n = 32 (50%)**	**n = 32 (50%)**	
Concurrent medical conditions/Clinical features			0.35
Anemia	0 (0)	2 (6.2%)	
Asthma, other: chlamydia (2009)	1 (3.1%)	0 (0)	
Asthma, other: Group B streptococcus carrier, maternal deficiency anemia, thrombocytopenia	1 (3.1%)	0 (0)	
Crohn’s disease	0 (0)	1 (3.1%)	
Diabetes - Type II	2 (6.2%)	1 (3.1%)	
Diabetes - Type II, morbid obesity, other: history of depression	1 (3.1%)	0 (0)	
Diabetes - Type II, other: left breast lump	1 (3.1%)	0 (0)	
Diabetes (gestational)	1 (3.1%)	3 (9.4%)	
Diabetes (gestational), obesity	1 (3.1%)	0 (0)	
Fatty liver	1 (3.1%)	0 (0)	
Hyperthyroidism	1 (3.1%)	0 (0)	
Migraines, urinary tract infection (UTI)	1 (3.1%)	0 (0)	
None	19 (59.4%)	24 (75%)	
Other: borderline gestational diabetes	1 (3.1%)	0 (0)	
Other: hepatitis C antibody = reactive	0 (0)	1 (3.1%)	
Other: history of cardiac surgery at birth, marginal cord insertion	1 (3.1%)	0 (0)	

The PE patients were diagnosed with preeclampsia characterized by both hypertension and proteinuria. As shown in Table 3, all of the 32 PE patients had both hypertension and proteinuria; 43.8% of them had headache; 21.9% of them had edema; and 25.0% of them had other additional symptoms. Other characteristics, including body mass index (BMI, prior to pregnancy), blood pressure (BP), protein/creatinine ratio (PCR) and pregnancy history are also shown in Table 4.

**Table 3:** PE patients’ presenting signs and symptoms

**Presenting signs and symptoms**	**Number (percentage)**
Hypertension	32 (100%)
Proteinuria	32 (100%)
Headache	14 (43.8%)
Edema	7 (21.9%)
Others	8 (25.0%)

PE, preeclampsia.

**Table 4:** PE patients’ clinical information

**Characteristics**	**Statistics**
BMI (prior to pregnancy) (kg/m^2^)	29.1 (23.0, 33.9)
Systolic blood pressure	146.0 (134.0, 157.5)
Diastolic blood pressure	85.5 (77.0, 94.5)
Protein/creatinine ratio (PCR) test results (mg/g)	803.5 (449.5 to 1492.0)
Prior history of preeclampsia	
Yes	3 (9.4%)
No	28 (87.5%)
Information not available	1 (3.1%)
Multiple gestation	
Yes	3 (9.4%)
No	29 (90.6%)
Number of abortions (induced or spontaneous)	
number = 0	21 (65.6%)
number = 1	5 (15.6%)
number = 2	2 (6.2%)
number = 3	2 (6.2%)
number = 4	0 (0%)
number = 5	1 (3.1%)
number = 6	1 (3.1%)
Number of full term pregnancies	
number = 0	22 (68.8%)
number = 1	2 (6.2%)
number = 2	3 (9.4%)
number = 3	3 (9.4%)
number = 4	1 (3.1%)
number = 5	1 (3.1%)
Number of premature pregnancies	
number = 0	27 (84.4%)
number = 1	4 (12.5%)
number = 2	1 (3.1%)
Smoking history	
Never	32 (100%)
Total number of pregnancies	
number = 1	14 (43.8%)
number = 2	4 (12.5%)
number = 3	5 (15.6%)
number = 4	4 (12.5%)
number = 5	2 (6.2%)
number = 6	1 (3.1%)
number = 7	0 (0%)
number = 8	0 (0%)
number = 9	0 (0%)
number = 10	1 (3.1%)
number = 11	1 (3.1%)
*In vitro* fertilization (IVF) utilized for this pregnancy	
No	32 (100%)

BMI, systolic blood pressure, protein/creatinine ratio (PCR) – Median (Interquartile range).

BMI, body mass index; PE, preeclampsia.

### Biomarker validation using preeclampsia and control maternal serum samples

To identify whether the PE serological protein panel could enable development of an immediate practical clinical tool, based on available ELISA assays, biomarker candidates, from expression multiplex analysis and two-dimensional gel profiling, were validated with available serum assays using PE (n = 32) and gestation age-matched control samples (n = 32). Detailed with whisker box and scatter plots in Additional file 2: Figure S1-(1 through 11), a total of 11 proteins were validated by ELISA assays (Mann–Whitney tests *p* value <0.05). Each validated biomarker’s median, mean and standard deviation of maternal serum abundance, in PE and control samples, are summarized in Table 5.

**Table 5:** Maternal serum levels of the validated PE biomarkers

**Analyte**	**PE trend**	**Unit**	**Early stage**				**Late stage**			
			**Normal**		**PE**		**Normal**		**PE**	
			**Median**	**Mean**	**Median**	**Mean**	**Median**	**Mean**	**Median**	**Mean**
			**(IQR)**	**(SD)**	**(IQR)**	**(SD)**	**(IQR)**	**(SD)**	**(IQR)**	**(SD)**
PIGF	↓	pg/ml	413.8	529.4	97.5	115.5	222.279	238.1095	184.488	202.6929
			(224.9, 685.2)	(432.0)	(51.6, 190.7)	(83.0)	(163.6, 289.9)	(111.5)	(113.2, 223.8)	(132.7)
sFlt-1	↑	pg/ml	1697.9	3034.0	19841.3	18646.3	5610.460	5531.241	14216.20	14414.28
			(1128.2, 4273.9)	(2578.7)	(15728.4, 21608.6)	(3582.5)	(4191.8, 6735.8)	(1811.9)	(12347.6, 19749.3)	(5575.3)
HPX	↑	ug/ml	1071.2	984.1	1382.8	1580.7	954.4	894.15	1482.0	1347.624
			(692.4, 1301.0)	(388.3)	(1173.6, 1787.0)	(546.5)	(538.0, 1131.6)	(331.5)	(1013.6, 1654.4)	(585.3)
ADAM12	↑	pg/ml	511.3	584.0	775.0	920.2	666.4185	703.6862	883.889	1345.369
			(437.7, 642.3)	(275.8)	(637.2, 1150.2)	(416.4)	(594.9, 791.8)	(217.2)	(626.7, 1367.6)	(1472.5)
APO C-III	↓	ng/ml	341.3	364.7	419.2	486.3	291.58	321.8587	453.789	585.7512
			(249.5, 422.4)	(153.4)	(357.3, 575.5)	(187.5)	(240.7, 345.7)	(126.7)	(308.9, 725.8)	(413.1)
HP	↓	ug/ml	1624.1	1718.0	1181.6	1482.7	1806.74	1750.72	985.616	1510.514
			(1216.0, 2274.1)	(764.1)	(684.6, 1794.1)	(1284.6)	(1190.1, 2163.1)	(684.1)	(592.0, 1880.8)	(1515.0)
A2M	↓	ug/ml	5796.4	5729.1	3365.1	4259.3	8141.38	7754.764	3435.427	4340.768
			(3501.2, 7737.6)	(3064.1)	(2648.3, 9959.0)	(2175.8)	(5300.6, 10234.1)	(3265.1)	(2343.7, 6752.9)	(2862.5)
APO-E	↓	ug/ml	290.6	364.4	138.8	215.9	398.0	377.9	147.2	150.0235
			(104.2, 519.0)	(301.5)	(63.0, 210.4)	(257.6)	(125.0, 478.4)	(236.3)	(60.4, 190.0)	(107.7)
APO A-I	↓	ng/ml	7980.1	8337.7	4945.356	4708.5	6253.298	6748.614	4724.142	5483.643
			(5775.7, 11076.6)	(3158.7)	(3892.8, 5824.6)	(1707.1)	(5624.1, 7881.8)	(2287.6)	(3138.6, 7075.3)	(3794.9)
RBP4	↓	ng/ml	38255.0	35180.4	38899.0	36931.7	41616.5	49253.5	33179	33897.47
			(29018.5, 40955.5)	(7031.1)	(33460.5, 39895.0)	(7307.5)	(38830.5, 44429.5)	(38081.6)	(29558, 37386)	(8499.8)
pikachurin	↓	ng/ml	601.8	659.1	293.261	327.8	536.551	536.551	317.657	317.657
			(563.8, 792.1)	(152.0)	(267.4, 367.8)	(117.5)	(459.2, 626.6)	(952.3)	(266.8, 409.7)	(623.5)

IQR: interquartile range; PE: preeclampsia; SD: standard deviation.

**Figure 2:**
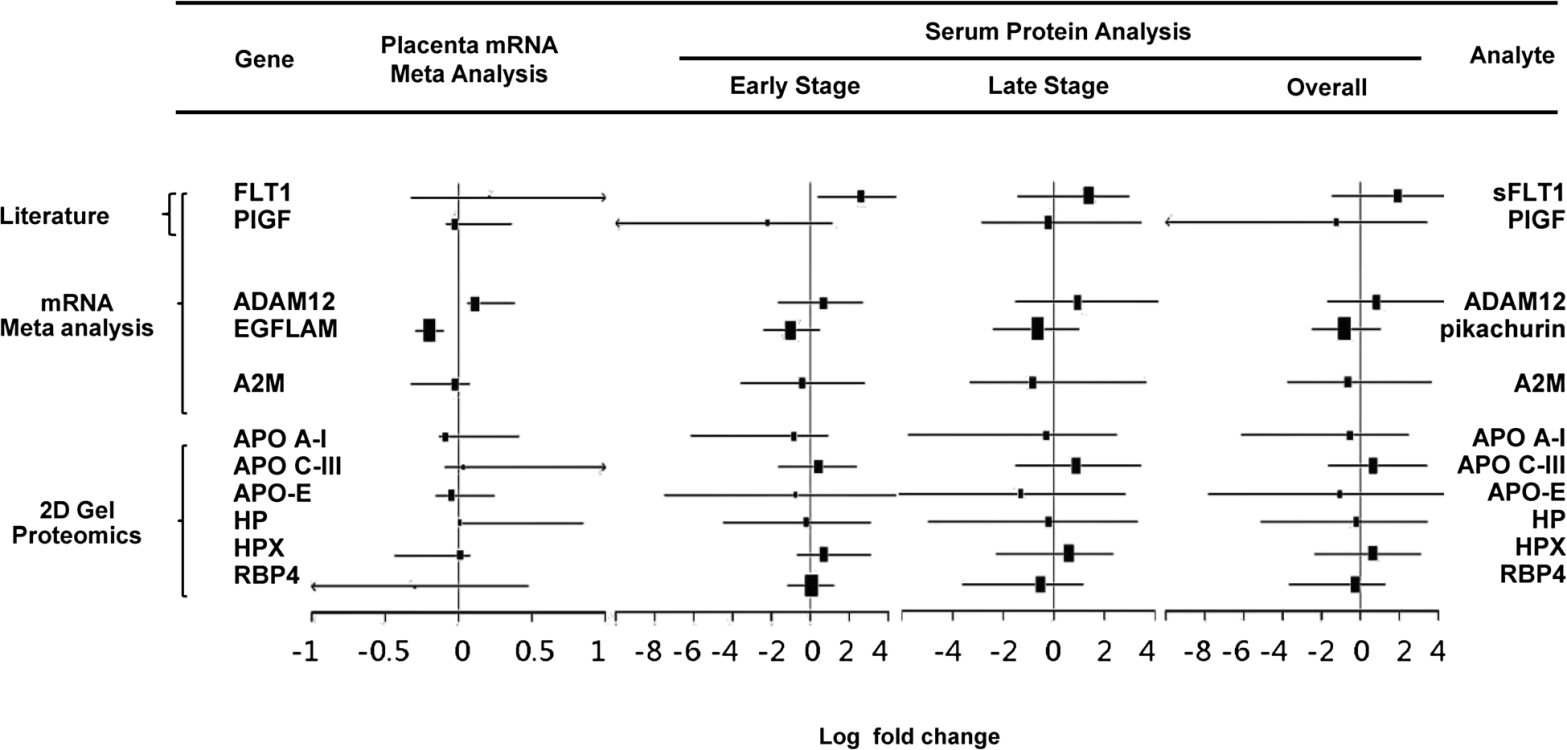
**Expression comparative analyses of PE biomarkers (PE versus controls).** Forest plot summarizes the results of placental mRNA expression multiplex analysis, and maternal sera analyte abundance quantification at different early and late gestational age weeks. Line plot represents 95% confidence intervals. PE, preeclampsia.

Forest plots (Figure 2) summarize the PE to control ratios of all 11 validated PE markers across placental expression multiplex analyses, and early and late gestation maternal serum analyses. The biomarkers derived from the proteomic and expression analyses consistently shared the same trend of up- or down-regulation between PE and control samples.

### Preeclampsia biomarker panel construction

**Figure 3:**
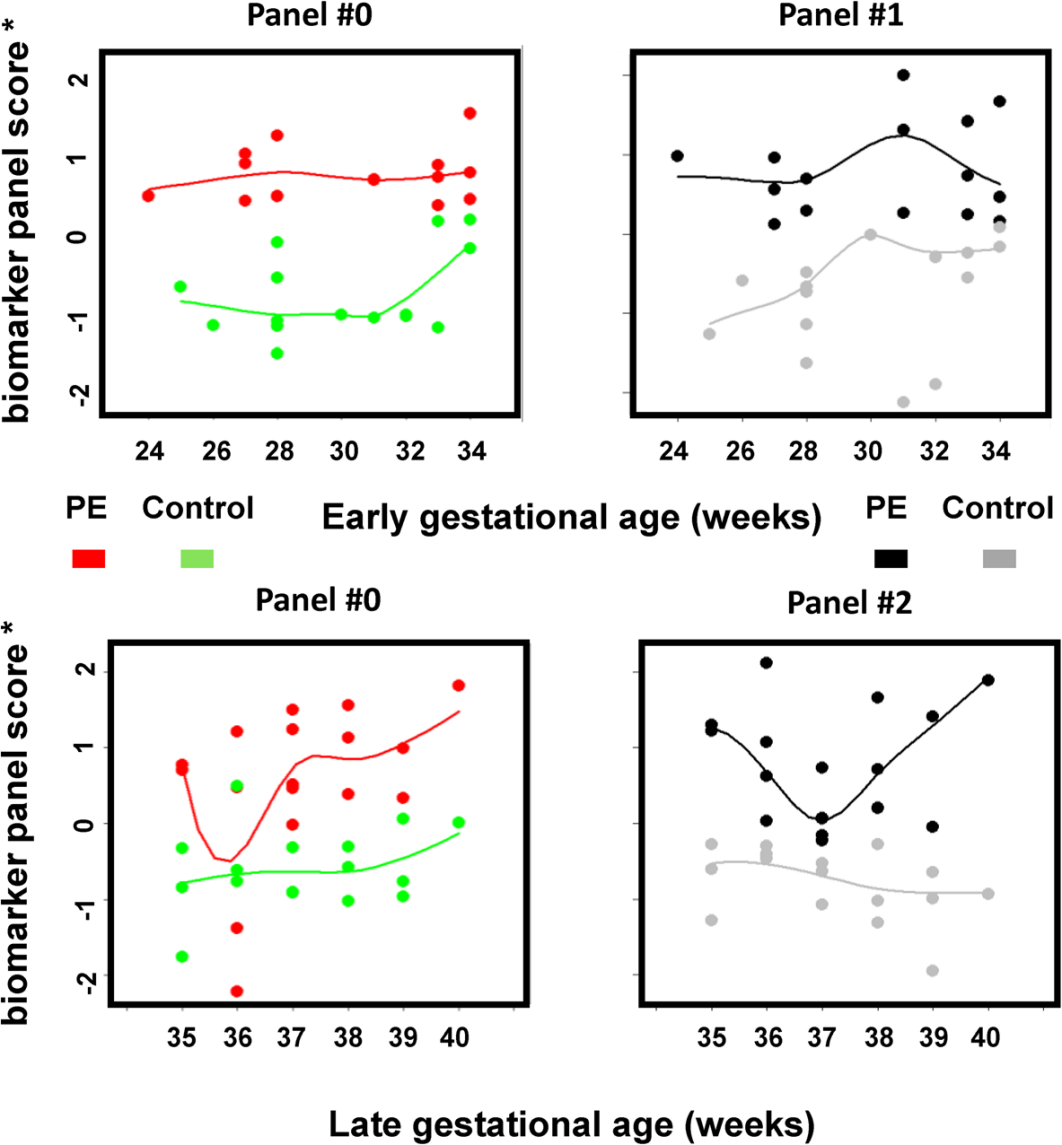
**Early or late onset biomarker panel scores were plotted as a function of the gestational age.** Different panel scores were scaled to the same scoring metric such that they can be directly compared. For either PE or control data points, a loess curve was fitted to represent the overall trend of biomarker scoring as a function of gestation. PE, preeclampsia.

Using data from the ELISA assays, we constructed different panels with various subsets of the assays. We sought to identify biomarker panels of optimal feature number, balancing the need for small panel size, accuracy of classification, goodness of class separation (PE versus control), and sufficient sensitivity and specificity. With the aim of developing a multiplexed antibody-based assay for PE diagnosis, we used a genetic algorithm method to construct biomarker panels from the nine validated PE protein biomarkers for early and late gestational age PE, comparing to the sFlt-1/PIGF ratio in assessing PE. The algorithm guided panel construction processes, leading to early and late gestational age biomarker panels, which had complete separation between PE and control subjects (Table 6). These chosen biomarker panels are non-redundant, indicating non-inclusive relationships. The sFlt-1/PIGF ratio’s PE assessment utility (panel 0: early onset, ROC area under the curve (AUC) 1.00, *p* value 4.35 × 10^-4^; late onset, ROC AUC 0.86, *p* value 2.94 × 10^-4^; Additional file 2: Figure S3), previously through the multicenter trial validation [24], was confirmed in this study and used as a benchmark for our newly derived biomarker panels. Panel 1 (early onset, ROC AUC 1.00, *p* value 1.43 × 10^-4^) has three proteins, HPX, APO A-I and pikachurin. Panel 2 (late onset, ROC AUC 1.00, *p* value 3.65 × 10^-5^) has six proteins, HPX, HP, APO C-III, APO A-I, RBP4 and pikachurin. To demonstrate the efficacy of the biomarker panel as a classifier for PE disease activity according to disease onset, the biomarker panel scores were plotted as a function of time of the gestational age (composite summary in Additional file 2: Figure S2, details shown in Figure 3). According to the scatter plot analysis, the performance of our early-onset PE biomarker panel was comparable to the sFlt-1/PIGF ratio. For gestational age >34 weeks samples, performance of our biomarker panel is better than the sFlt-1/PIGF ratio that has several errors of diagnosis around week 36. Among the early and late gestational age biomarker panels, HPX, APO A-I, and pikachurin are present in both panels, indicating their critical role in the diagnosis.

**Table 6:** Biomarker panels integrating maternal serum levels of the validated PE biomarkers

**PE onset**	**Early**		**Late**	
Panel	0	1	0	2
sFlt-1^a^	+	-	+	-
PIGF	+	-	+	-
HPX^a^	-	+	-	+
FT	-	-	-	-
ADAM12^a^	-	-	-	-
HP	-	-	-	+
A2M	-	-	-	-
APO-E	-	-	-	-
APO C-III^a^	-	-	-	+
APO A-I	-	+	-	+
RBP4	-	-	-	+
HB	-	-	-	-
FGA	-	-	-	-
pikachurin	-	+	-	+
Panel size	2	3	2	6
ROC AUC	1.00	1.00	0.86	1.00
*p* value	4.35E-4	1.43E-4	2.94E-4	3.65E-4

^a^Indicates biomarkers up-regulated in PE. Panel 0 is the benchmark panel sFlt-1/PIGF ratio; ‘+’, selected in panel; ‘-‘, unselected.

AUC: area under the curve; PE: preeclampsia; ROC: receiver operating characteristic.

### Pathway analysis of PE biomarkers

We analyzed the validated biomarkers that are significantly differentially expressed in PE as a composite, using Ingenuity Pathway Analysis software (IPA version 7.6, Ingenuity Systems, Inc., Redwood City, CA, USA). In addition to the heme/hemoglobin degradation pathway revealed during our multi-’omic’ discovery effort, our pathway analysis led to the identification of the following statistically significant canonical pathways which may play important roles in PE pathophysiology: liver X receptor (LXR)/retinoid X receptor (RXR) activation, *p* value 5.13 × 10^-9^; atherosclerosis signaling, *p* value 5.01 × 10^-7^; IL-12 signaling and production in macrophages, *p* value 8.51 × 10^-7^; acute phase response signaling, *p* value 1.91 × 10^-6^; production of nitric oxide and reactive oxygen species in macrophages, *p* value 2.82 × 10^-6^; clathrin-mediated endocytosis signaling, *p* value 2.88 × 10^-6^; farnesoid X receptor (FXR)/RXR activation, *p* value 2.04 × 10^-5^; hepatic fibrosis/hepatic stellate cell activation, *p* value 2.88 × 10^-3^; phosphatidylethanolamine biosynthesis II, *p* value 1.05 ×10^-2^; coagulation system, *p* value 2.04 × 10^-2^; growth hormone signaling, *p* value 4.27 × 10^-2^; reelin signaling in neurons, *p* value 4.57 × 10^-2^; and VEGF family ligand-receptor interactions, *p* value 4.79 × 10^-2^.

## Discussion

We have applied a multi-’omics’ approach to develop validated PE biomarkers, integrating discoveries from placental mRNA expression multiplex analysis and depleted serological proteome two-dimensional gel comparative profiling. Comparing PE and control sera with commercially available ELISA assays, we have validated 11 protein markers, including sFlt-1 and PIGF, and found that our identified PE biomarkers were superior over the sFlt-1/PIGF ratio in diagnosing PE. The concept of combining a transcriptomic approach in placenta tissue with a proteomic approach in serum is novel. It combines the advantages of a study in tissue which is closer to the focus of the pathophysiology with those of a study in serum which is more appropriate for clinical use. Taking proteins that have been discovered from the discovery phase to an ELISA-based validation phase makes the findings of this study translatable into clinical practice.

When comparing the discoveries from expression multiplex analysis and two-dimensional gel serum proteomics, only A2M showed up in both analyses. This could be due to the following reasons: (1) the discordant expression of protein and mRNA as previously characterized [38-41]; (2) the lack of translation of the placental expression into circulation protein level abundance; and (3) two-dimensional gel technology detection limit of 0.5 to 5 ng. The optimized two-dimensional gel technique has a dynamic range of approximately five orders of magnitude in protein concentration [42], whereas serological protein concentrations vary over approximately ten orders of magnitude, with the highest concentrations reaching mg/mL [43]. Even with the depletion step, protein detection by our two-dimensional gel is limited to proteins whose serological concentrations are >10 ug/mL, clearly influencing the composition of the protein biomarkers we detected. In addition, potentially informative low molecular weight proteins may bind to albumin and, thus, be removed at the depletion step [44], which could be a potential disadvantage. Thus, candidates with pg/mL concentration, for example, sFlt-1 and PIGF, would not be found when applying the two-dimensional gel serum proteomics based approach. Publicly available genome-wide gene expression data on disease tissues can be effectively mined to provide significant synergies to complement our two-dimensional serum proteomics efforts to unveil differential PE biomarker candidates of low serum abundance (pg/mL). Notably, our productive PE discovery efforts support the notion that the multi-’omics’ approach for biomarker analyses are comprehensive, complementary and effective in identifying candidates of a broad dynamic range of serological protein expression, varying from pg/mL to ug/mL.

As summarized in Figure 2, the validated biomarkers’ placental expression, and the early and late gestation maternal serum analyses revealed a similar trend of up- or down-regulation between PE and control samples. However, our study did not explore the extent (percentage) of the contribution by the placenta or other maternal cells to the overall differential serum expression between PE and control subjects. Future expression analysis is needed to characterize the tissue expression pattern of these PE markers and their expression kinetics as a function of the gestational age to understand the tissue specific expression contribution to the differential serum expression pattern observed in this study.

Additional pathway analyses of the protein markers corroborate growing evidence implicating roles for the lipid homeostasis, IL-12 and coagulation canonical pathways in PE pathophysiology. The LXR/RXR activation pathway was identified as the most significant pathway. This supports recent findings [45] that PE is associated with hyperlipidemia and that the regulators of lipid homeostasis are important in the PE pathophysiology. The previous evidence [46-48] of IL-12, in PE patients, with less activity in placenta and more abundance in sera was reflected as in line with our PE biomarker panel pattern pathway analysis.

A previous multicenter case–control study [24] with an automated assay, demonstrating the utilities of sFlt-1 and PIGF for PE assessment, reported serum abundance of sFlt-1 (PE: 12,981 ± 965 versus control: 2,641 ± 100.5 pg/mL) and PIGF (PE: 76.06 ± 10.71 versus control: 341.5 ± 13.57 pg/mL). Although with greater variation, possibly due to different sample cohorts or assay platforms, the trend of alteration reflected in our results, sFlt-1 (PE: 16,398.02 ± 5,142.32 versus control: 4,282.63 ± 2,532.90 pg/mL) and PIGF (PE: 161.83 ± 118.98 versus control: 383.75 ± 343.84 pg/mL) was in line with their report. As shown in Additional file 2: Figure S1 and summarized in Additional file 1: Table S2, sFlt-1 and PIGF protein abundance differs significantly between early and late gestational age samples in both normal (*p* value: sFlt-1 0.003, PIGF 0.020) and PE (*p* value: sFlt-1 0.017, PIGF 0.022) groups. Our biomarker [see Additional file 1: Table S2] RBP4 (*p* value: normal 0.029, PE 0.176), ADAM12 (*p* value: normal 0.035, PE 0.777) and pikachurin (*p* value: normal 0.049, PE 0.502) differs marginally between early and late gestational age samples in normal (*p* value <0.05) but not in PE (*p* value >0.05) groups. For HPX, APO C-III, HP, APO-E and APO A1, there was no significant difference (*p* value >0.05) between early and late gestation sera in both normal and PE groups. The sFlt-1/PIGF ratio was found to be important for the prediction of both preeclampsia and intrauterine growth restriction (IUGR) [49]. Therefore, the previous observation of the sFlt-1 and PIGF expression difference between early and late onset cases may be due to the recruited sample difference between early (both IUGR and early PE) and late (PE only) cases. Given that our sample cohort excluded IUGR, the serum markers identified in this study may be more specific to PE rather than to both IUGR and PE. To summarize, our results here indicate that sFlt-1 and PIGF are regulated during placental development as a function of gestation, and differential expression between PE and control might be due to placental adaptation during PE. The PE biomarkers found in this study are not significantly different between early and late gestation in either PE or control sera. Therefore, their differential expression in PE might directly gauge the disease activity of PE and disease development or reflect features that are present at fairly advanced stages of the pathogenesis, for example, proteinuria and high blood pressure.

Our genetic algorithm-based biomarker panel construction led to final early and late gestational age biomarker panels for PE assessment. Compared to the benchmark sFlt-1/PIGF ratio in PE assessment, our biomarker panels perform comparably during early gestational age but clearly outperform at later gestational weeks. Although the sFlt-1 and PIGF imbalance used for PE diagnosis has been demonstrated, there is mounting evidence to support the notion that normal sFlt-1 and PIGF expression actually characterizes healthy pregnancies [50]. Therefore, sFlt-1 and PIGF may really be general markers for failed pregnancies, for example, ectopic pregnancies, missed abortions, rather than specific to PE. Our multi-’omics’ approach discovered panels of multiple biomarkers, reflecting the multifaceted aspects of PE disease, and have the potential both to provide a definitive diagnosis of PE patients and to be used to monitor disease progression.

We also recognize several limitations to our study. Samples were collected after the clinical diagnosis of PE with disease onset. The outcome information after the sample collection, including the time of delivery, and the birth weight and growth percentile of the babies, is not available. Therefore, the biomarker panels’ utility in risk patient identification remains to be demonstrated. Nevertheless, confirmatory diagnosis is also valuable as it has the benefit of objective diagnosis, reducing over and under diagnoses. To translate our innovative PE markers to clinics, a clinical trial of the prospective cohort design is needed. As one of the limitations of this study, we used commercially available ELISA kits, the antibodies of which may cross-react with other homologous proteins. For example, the R&D sFlt-1 ELISA kit antibodies can recognize both sFlt-1 and full trans-membrane VEGF-R1, as well. To support future prospective trials to test the clinical utility of our PE panel, analyte specific antibody reagents may need to be developed.

Although preeclampsia is diagnosed when a pregnant woman develops both elevated blood pressure and proteinuria, these symptoms tend not be specific and preeclampsia can be asymptomatic as well. Therefore, the clinical definition alone is insufficient to predict adverse maternal and/or neonatal outcomes [51,52] caused by preeclampsia. Previous prospective cohort studies have found the utility of the elevated sFlt1/PIGF ratio in the prediction of the subsequent adverse maternal and prenatal outcomes within two weeks [53]. The scatter plot analysis (Figure 3) as a function of gestational age suggests that, for our early onset panel, the best performance in PE assessment was obtained near 24 to 25 weeks when comparing to gestation towards 34 weeks. Certain changes in our biomarker panel of serum protein profiles may occur at the first trimester and in advance of clinically-detectable PE disease activity. Thus, we hypothesize that our PE biomarkers can predict impending PE disease activity, and/or adverse outcomes in pregnant women with suspected preeclampsia, especially in a pre-specified group of patients presenting at less than 24 weeks gestation. To test these hypotheses, future prospective cohort studies will be required to address the potential clinical usefulness of our PE biomarkers in predicting impending PE or adverse maternal and/or neonatal outcomes.

## Abbreviations

A2M: Alpha-2-macroglobin; ADAM12: A disintegrin and metalloproteinase domain-containing protein 12; ADRP: Adipophilin; APO: A-I apolipoprotein A-I; APO: C-III apolipoprotein C-III; APO-E: Apolipoprotein –E; AUC: Area under the curve; BMI: Body mass index; CCL2: Chemokine (C-C motif) ligand 2; CTSB: Cathepsin B; CTSC: Cathepsin C; EGFLAM: Pikachurin; ELISA: Enzyme-linked immunosorbent assay; FDR: False discovery rate; HMOX1: Heme oxygenase 1; HP: Haptoglobin; IGFBP7: Insulin-like growth factor binding protein 7; IQR: Interquartile range; IRB: Institutional Review Board; IVF: *in vitro* fertilization; KNG1: Kininogen 1; KRT33A: Keratin 33A; KRT40: Keratin 40; PCR: Protein/creatinine ratio; PE: Preeclampsia; PIGF: Placental growth factor; PPBP: Pro-platelet basic protein; RBP4: Retinol-binding protein 4; ROC: Receiver operating characteristic; SD: Standard deviation; sFlt-1: Soluble fms-like tyrosine kinase.

## Competing interests

XBL and AJB are co-founders and equity holders of Carmenta Bioscience, which is currently developing a commercial serum-based diagnostic test for preeclampsia. The other authors declare that they have no competing interests.

## Authors’ contributions

XBL, DKS and AJB designed the study and wrote the paper. LL, AAM and DJL performed the gene expression multiplex analysis. TY and GC performed two-dimensional gel analysis and ELISA validation. JJ, QW and BJ performed the statistical analysis. All authors read and approved the final manuscript.

## Pre-publication history

The pre-publication history for this paper can be accessed here:

http://www.biomedcentral.com/1741-7015/11/236/prepub

## Supplementary Material

Additional file 1: Table S1 Expression data sets used for multiplex meta analysis based PE marker discovery. **Table S2.** Comparison of biomarker’s abundances at early and late gestational age time points. Click here for file

Additional file 2: Figure S1 Boxplot display and scatter plot of biomarker distributions at different gestation in PE and control groups. Horizontal box boundaries and midline denote sample quartiles. **Figure S2.** Composite overlay of different biomarker panels’ loess fitted lines for both PE and control subjects as a function of gestation. **Figure S3.** The performance, gauged by ROC analyses, of PE serum protein biomarker panel 0, 1, and 2 in discriminating PE and control subjects. Click here for file
