# Supplementary material for: Integrating multiple ‘omics’ analyses identifies serological protein biomarkers for preeclampsia
Source: BMC Med. 2013 Nov 6;11:236. doi: 10.1186/1741-7015-11-236 (PMC4226208; doi:10.1186/1741-7015-11-236)
Supplement: Additional file 1: Table S1 — Expression data sets used for multiplex meta analysis based PE marker discovery. Table S2. Comparison of biomarker’s abundances at early and late gestational age time points. [file 1741-7015-11-236-S1.pdf]

Supplementary Table 1. Expression data sets used for multiplex meta analysis based PE marker discovery.

Dataset	Title	Tissue	Cases	Controls
Nishizawa et al Placenta 2007	Differentially Expressed Genes in Placental Tissue of Severe Preeclampsia	placenta	10	4
Tsai et al Placenta 2011	Transcriptional Profiling of Human Placentas from Pregnancies Complicated by Preeclampsia	placenta	23	37
Nishizawa et al 2011	Gene expression profiling for placentas from pre-eclamptic, unexplained FGR and normal pregnancies	placenta	8	8
Winn et al Endocrinology 2009	Severe Preeclampsia-Related Changes in Gene Expression at the Maternal-Fetal Interface	placenta	12	11
Sitras et al Placenta 2009	Placental gene expression in severe preeclampsia	placenta	17	26
Founds et al Placenta 2009	Chorionic villus sampling (CVS) microarray in preeclampsia	CVS	4	8
Roten et al MolHumRep 2011	Transcription profiling of human decidua basalis to identify pre-eclampsia susceptibility genes	Decidua basalis	37	58
		**Total**	111	152

Supplementary Table 2. Comparison of biomarker's abundances at early and late gestational age time points

Analyte	Control		PE	
	Fold*	*p* value**	Fold*	*p* value**
**PIGF**	0.449787	0.020445	1.754707	0.021946
**sFlt-1**	1.823071	0.002984	0.773039	0.017316
**HPX**	0.908643	0.509422	0.852538	0.433073
**ADAM12**	1.204841	0.034792	1.462044	0.776988
**APO C-III**	0.882512	0.445036	1.204613	1
**HP**	1.019037	0.780443	1.018754	0.940656
**A2M**	1.353563	0.079568	1.019117	0.852335
**APO-E**	1.036976	0.668931	0.694897	0.820737
**APO A-I**	0.80941	0.146736	1.164625	0.911083
**RBP4**	1.400028	0.028797	0.917843	0.176456
**pikachurin**	0.832876	0.047833	1.070773	0.501947

*Fold was calculated by the ratio of the medians of early and late gestational age samples' assayed biomarker abundances
** *p* value: Mann-Whitney U test
