# Supplementary material for: Integrating multiple ‘omics’ analyses identifies serological protein biomarkers for preeclampsia
Source: BMC Med. 2013 Nov 6;11:236. doi: 10.1186/1741-7015-11-236 (PMC4226208; doi:10.1186/1741-7015-11-236)

Supplementary Figure 1-1

sFlt-1 Validation

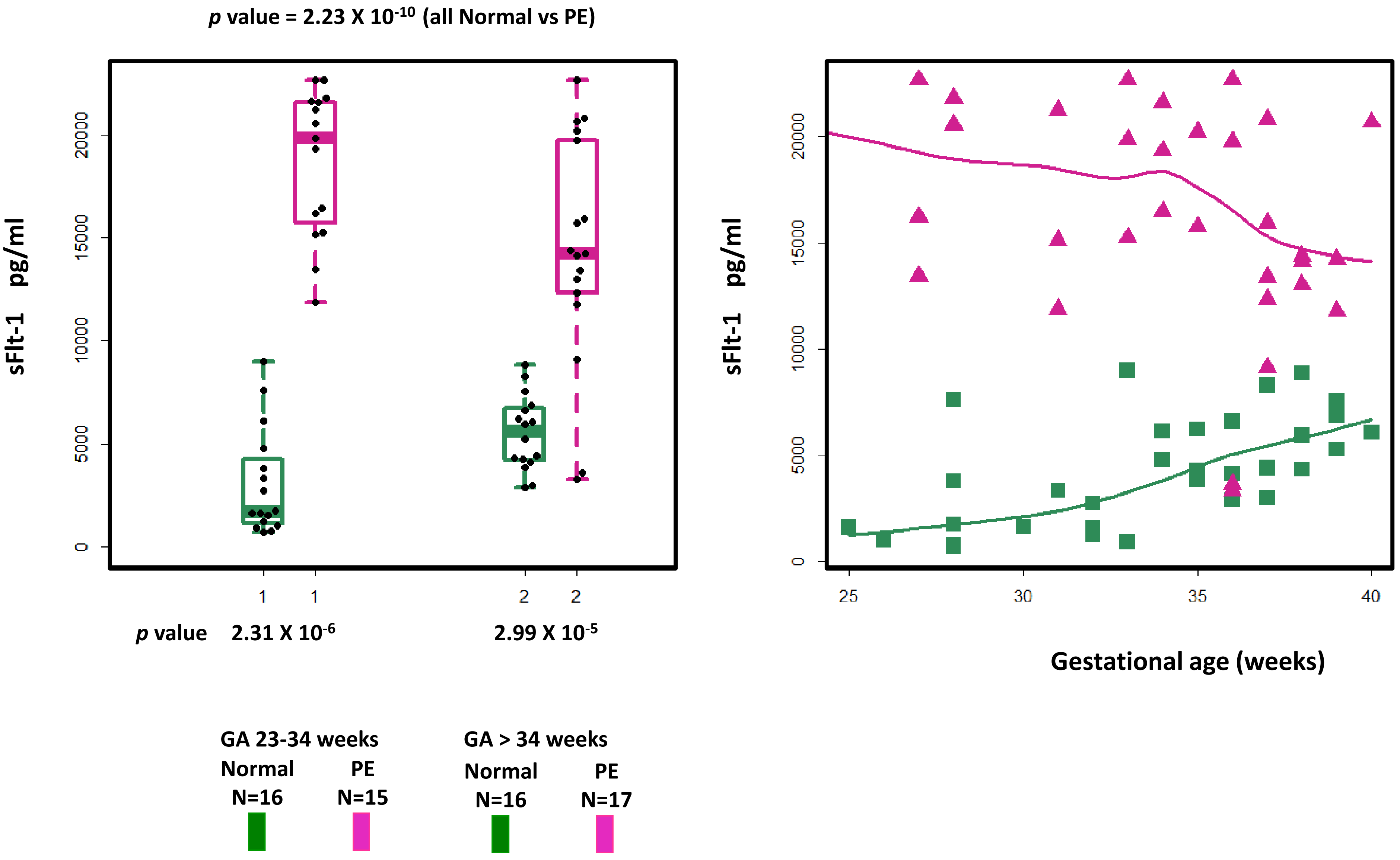

Supplementary Figure 1-2

PIGF Validation

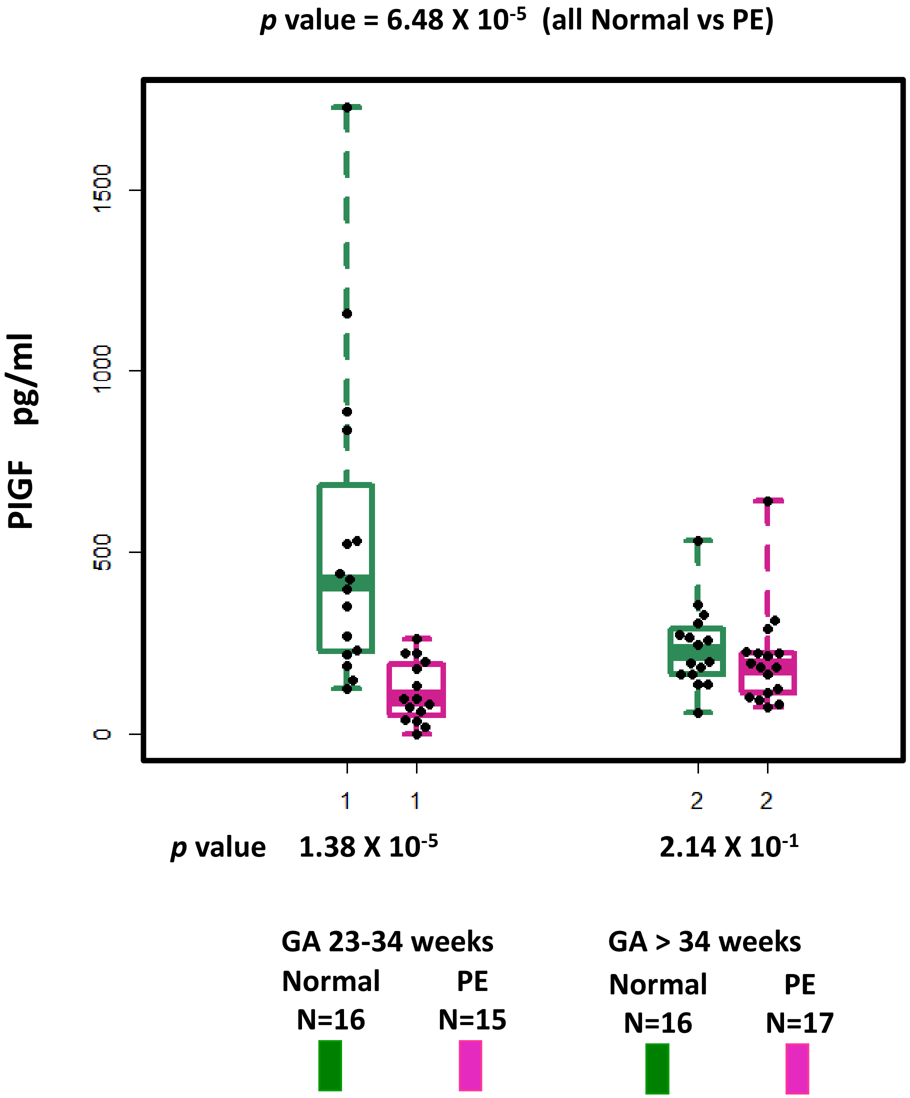

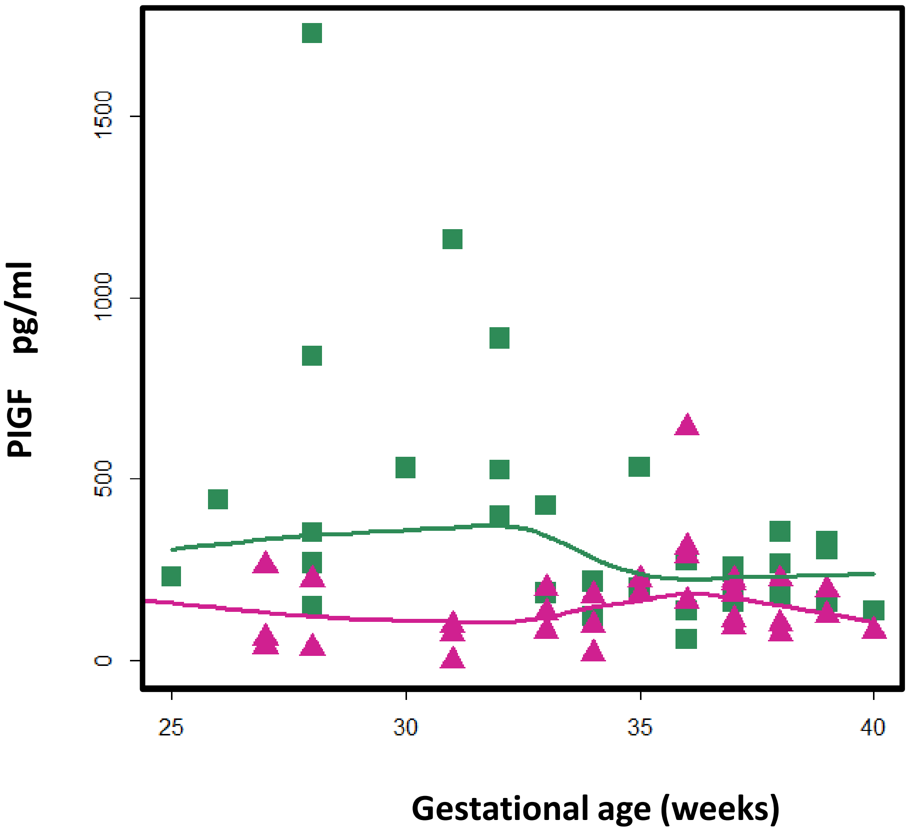

HPX Validation

p value = 8.581 X 10^{-5} (all Normal vs PE)

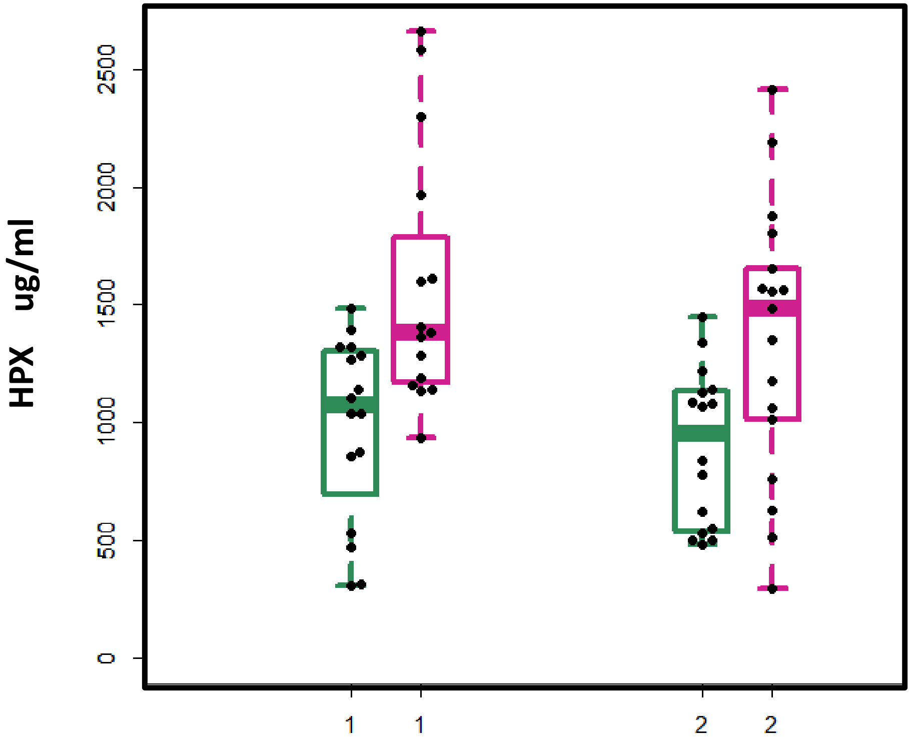

p value 2.84 X 10^{-3} 1.51 X 10^{-2}

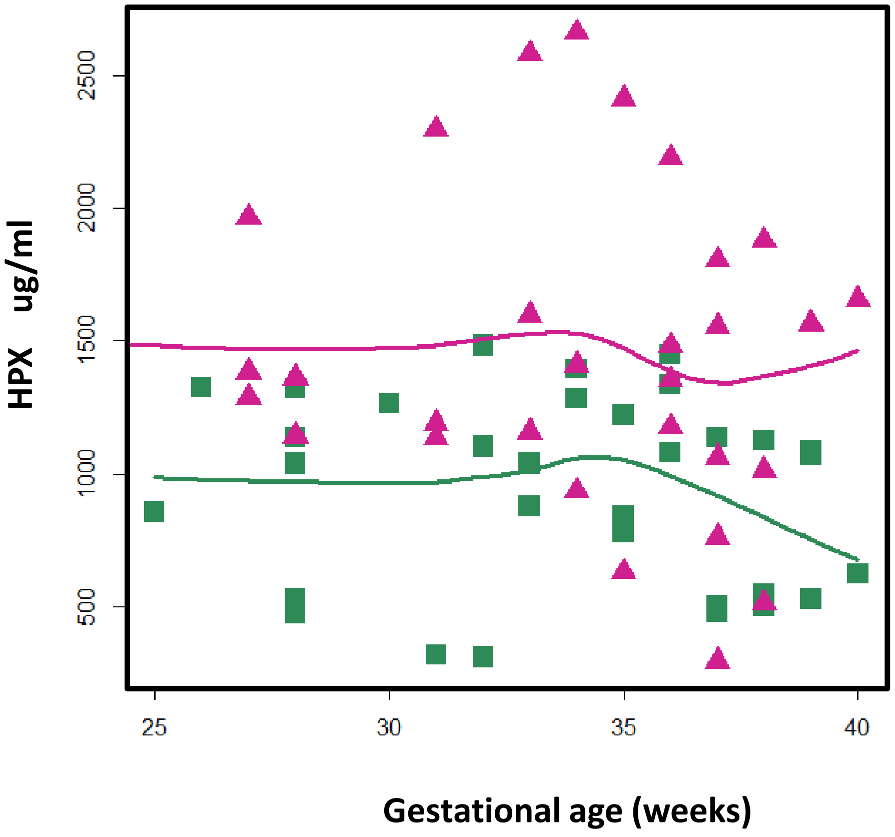

GA 23-34 weeks		GA > 34 weeks	
Normal N=16	PE N=15	Normal N=16	PE N=17

Supplementary Figure 1-4

ADAM12 Validation

p value = 1.50 X 10^{-3} (all Normal vs PE)

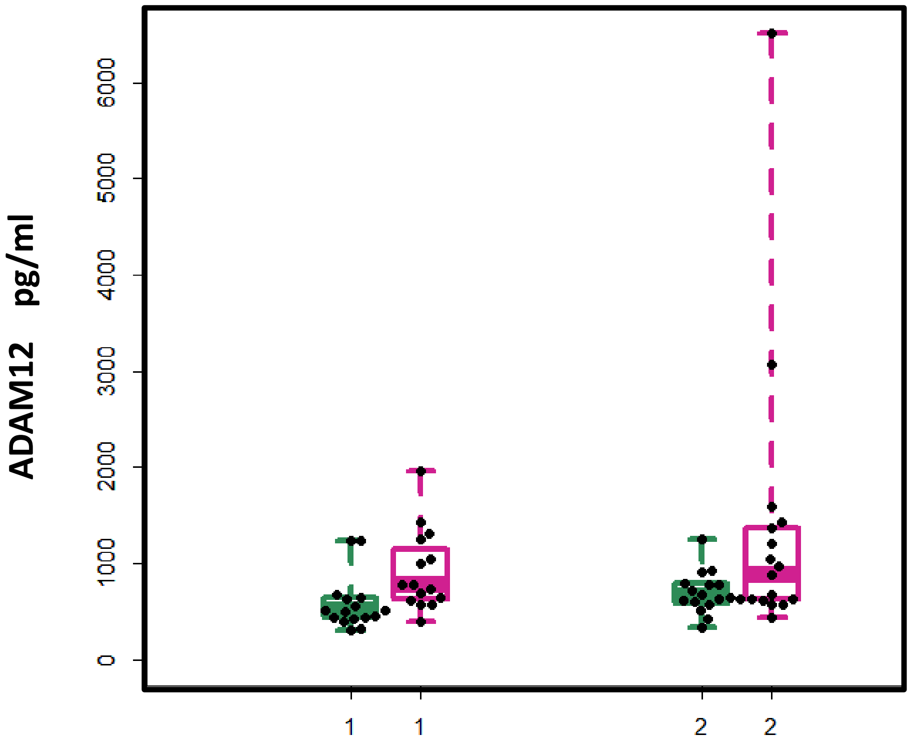

p value 2.01 X 10^{-3} 1.27 X 10^{-1}

GA 23-34 weeks		GA > 34 weeks	
Normal N=16	PE N=15	Normal N=16	PE N=17

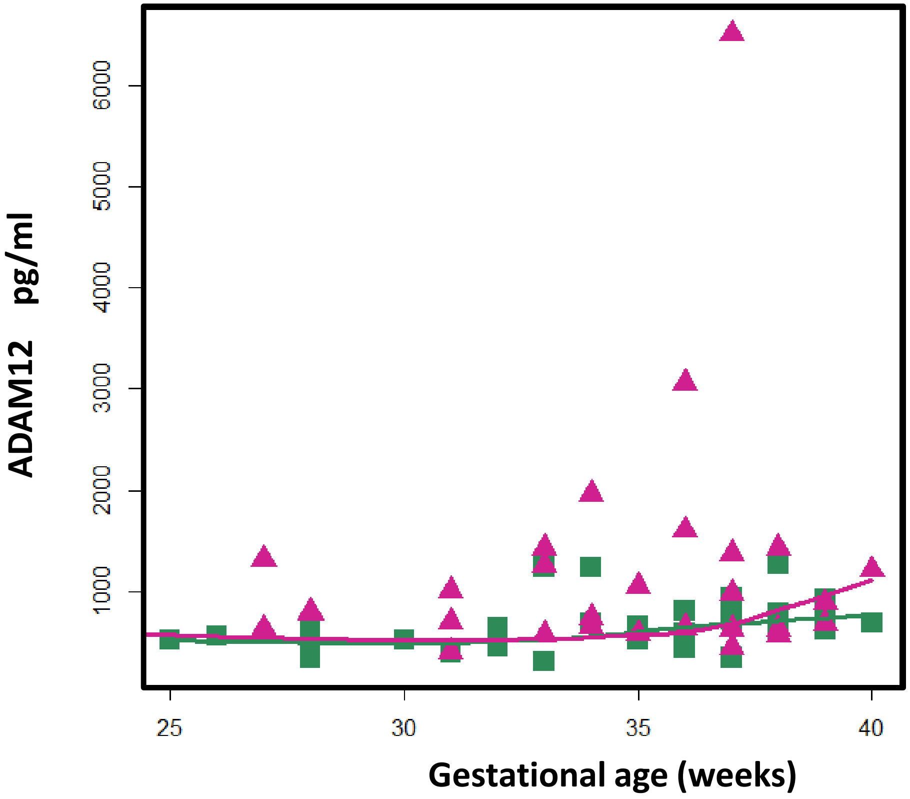

HP Validation

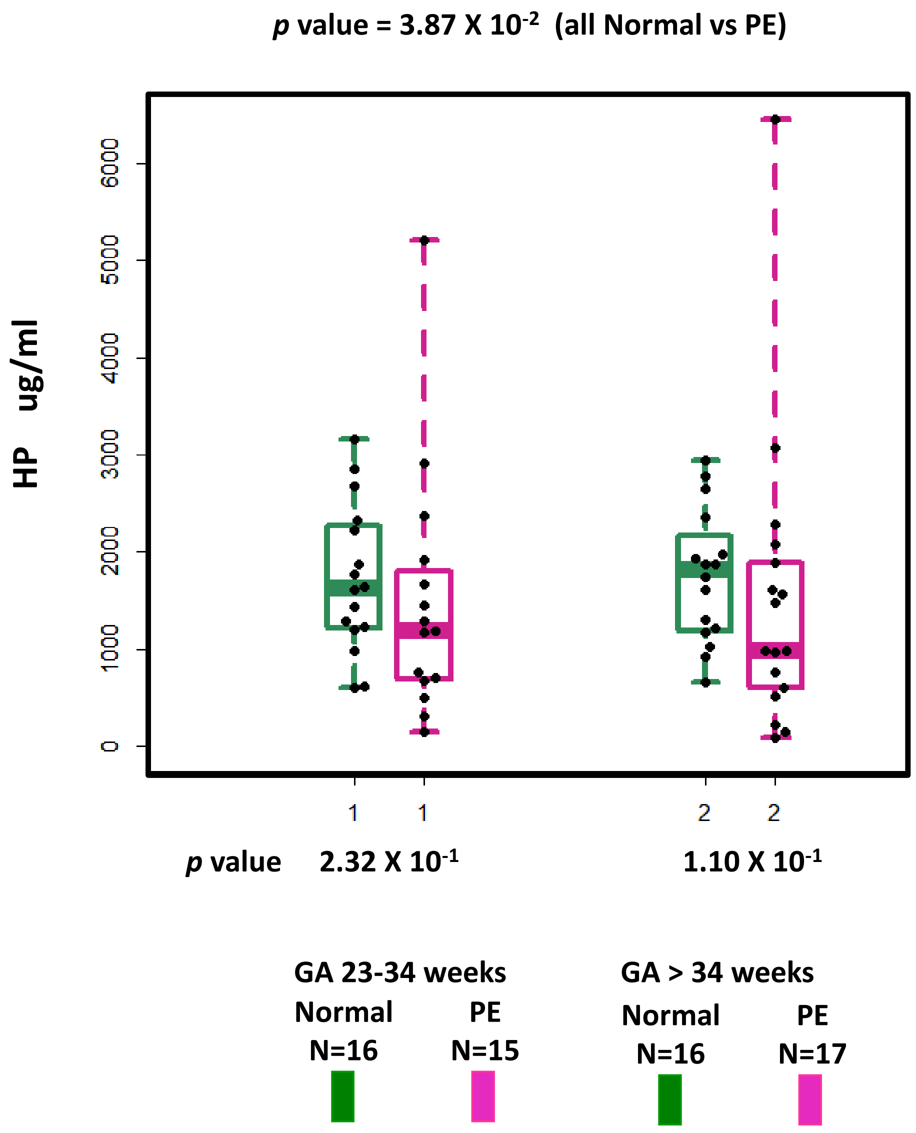

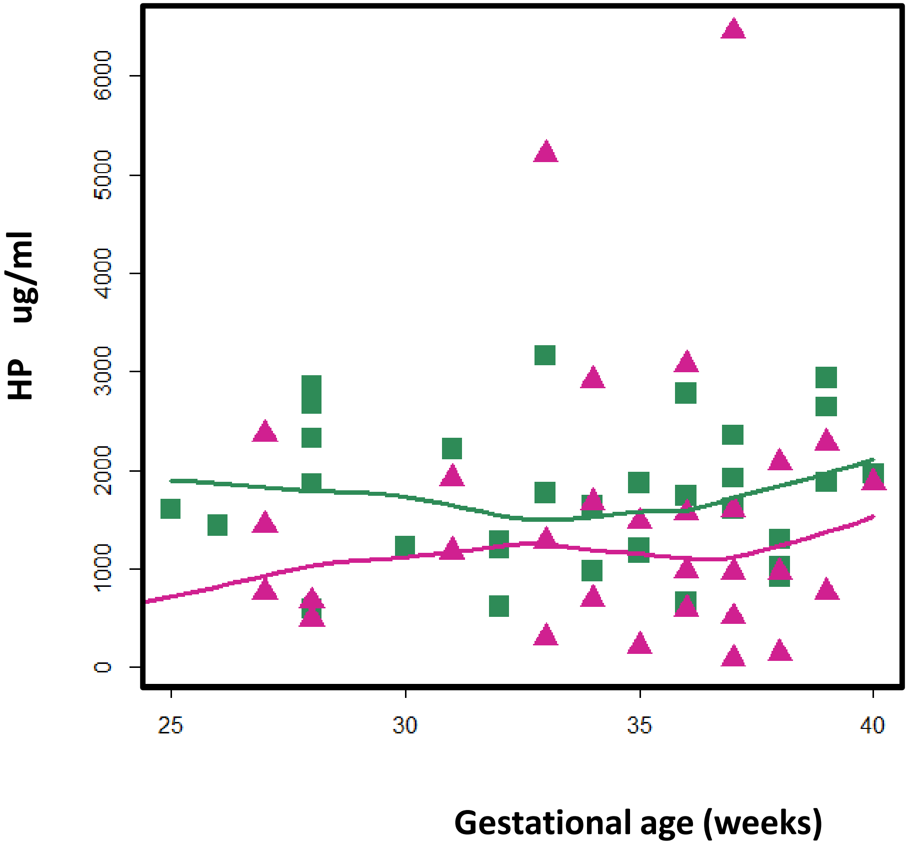

Supplementary Figure 1-6

A2M Validation

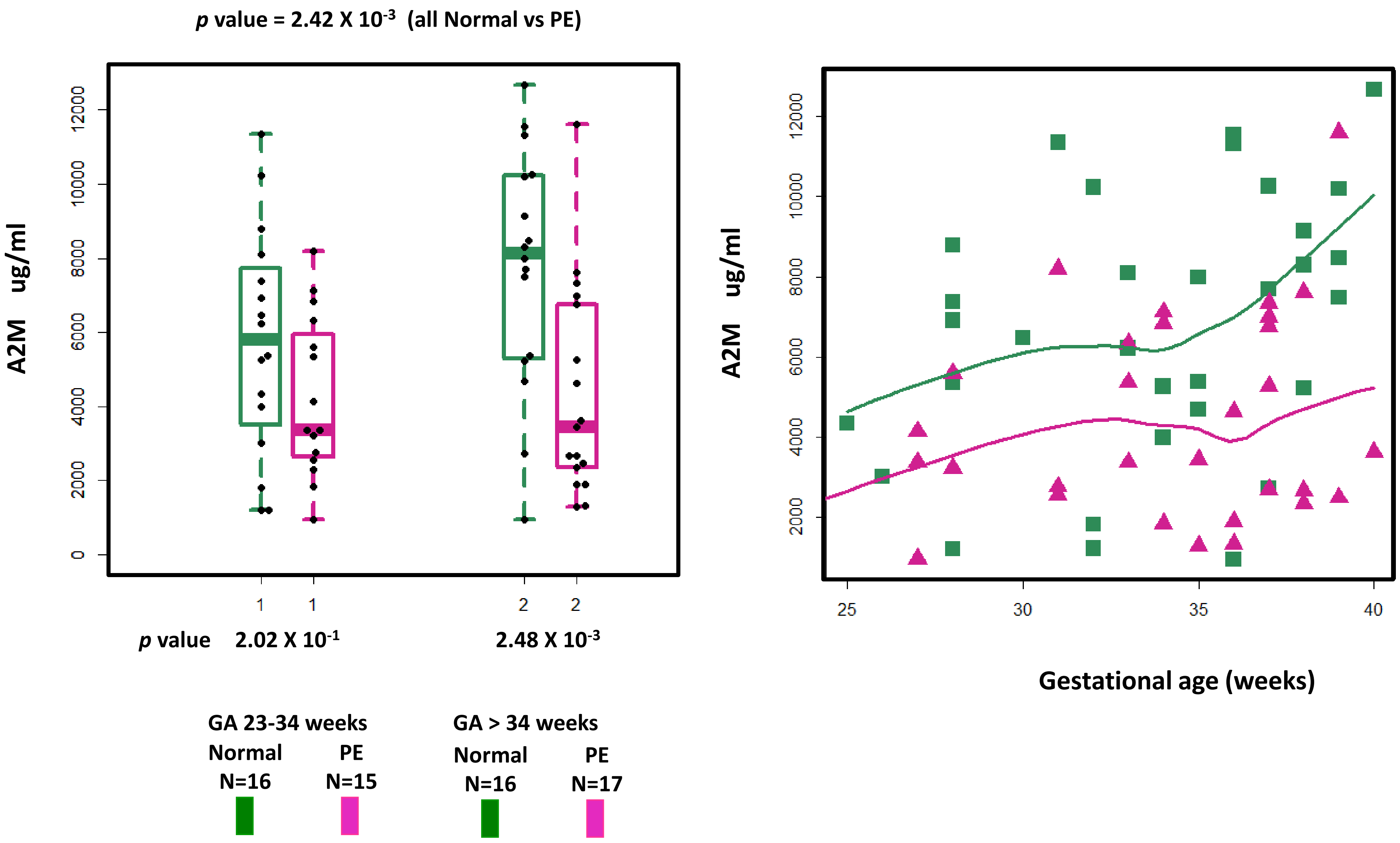

APO-E Validation

p value = 2.25 X 10^{-3} (all Normal vs PE)

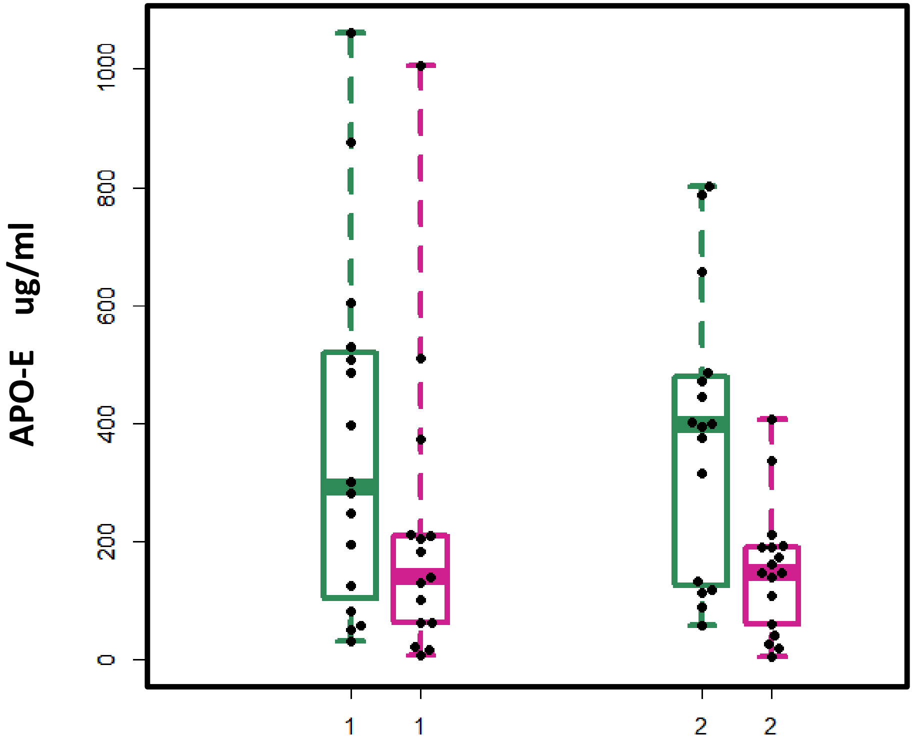

p value 1.10 X 10^{-1} 1.00 X 10^{-2}

GA 23-34 weeks		GA > 34 weeks	
Normal	PE	Normal	PE
N=16	N=15	N=16	N=17

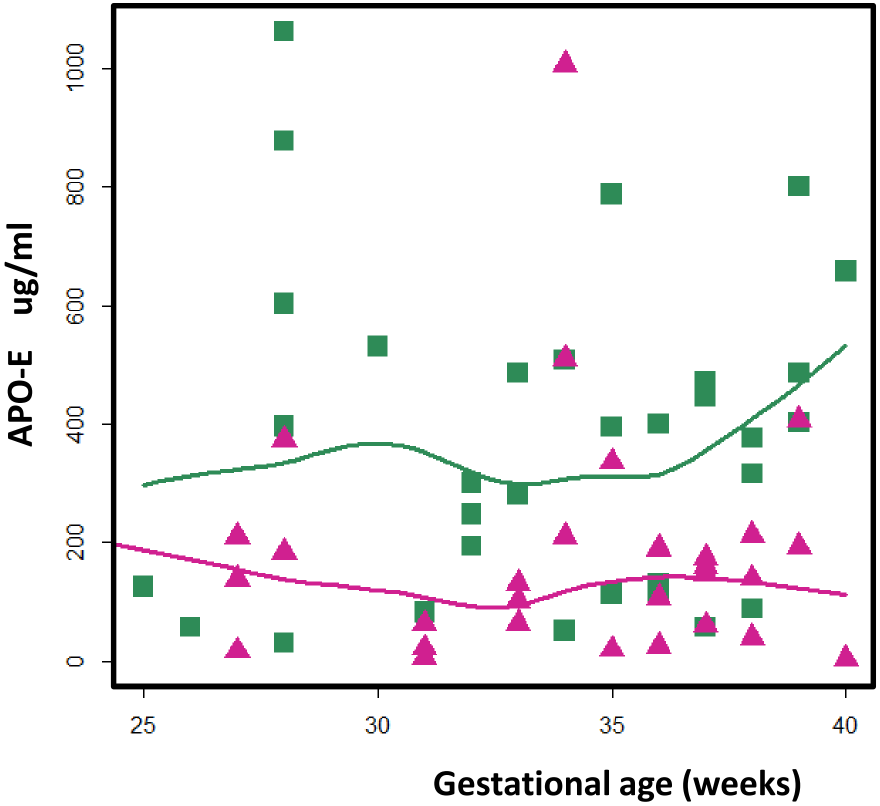

Supplementary Figure 1-8

APO C-III Validation

p value = 9.46 X 10^{-4} (all Normal vs PE)

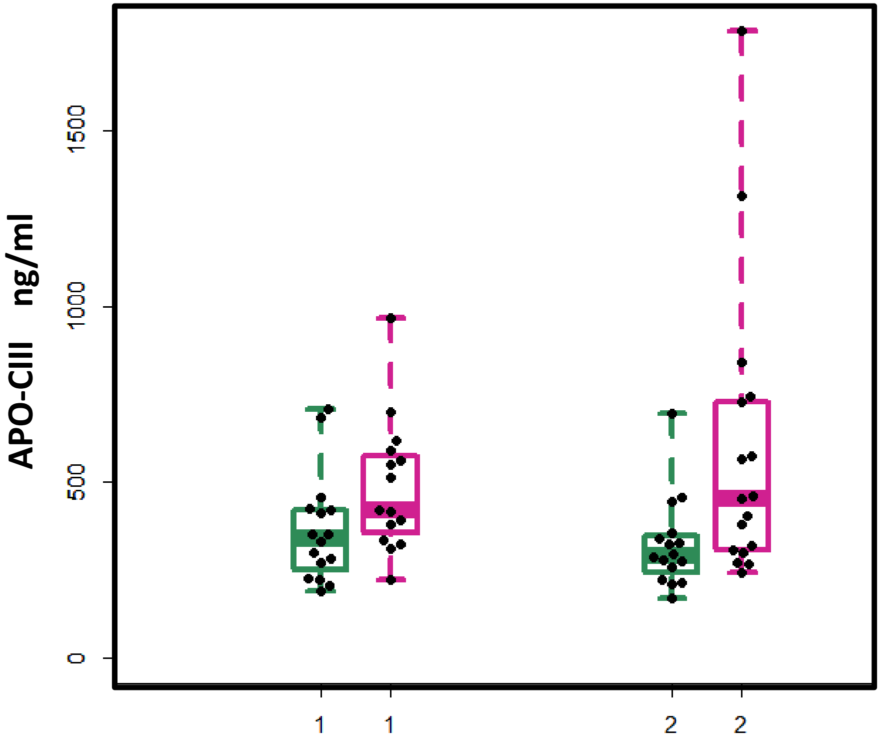

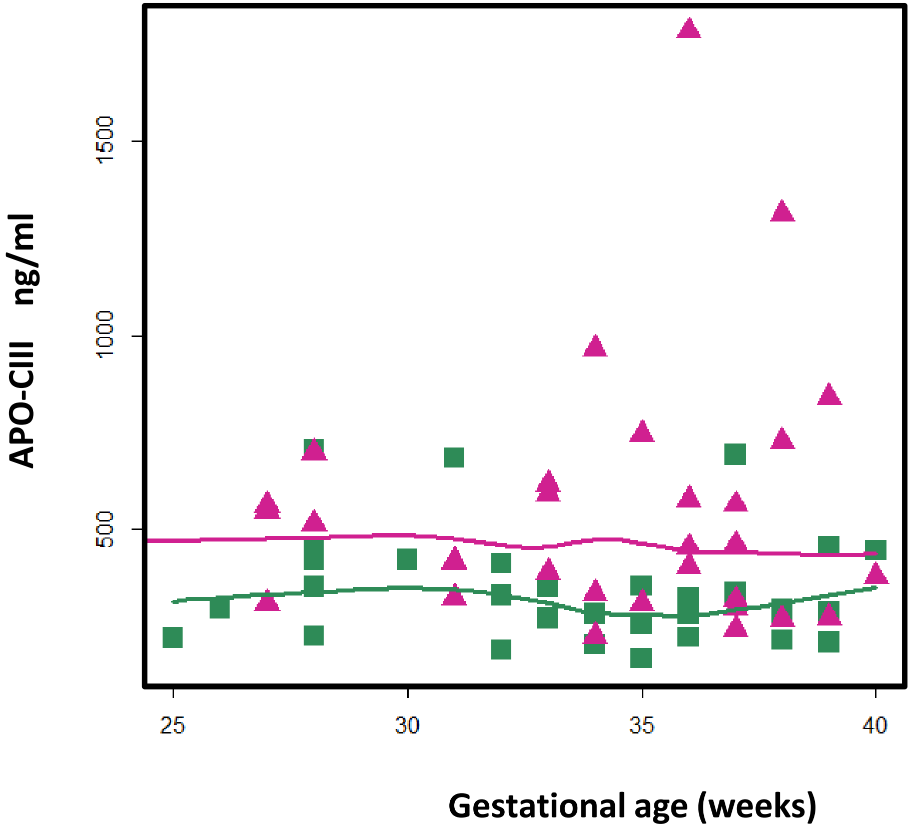

p value 5.97 X 10^{-2} 1.09 X 10^{-2}

GA 23-34 weeks		GA > 34 weeks	
Normal N=16	PE N=15	Normal N=16	PE N=17

Supplementary Figure 1-9

APO A-I Validation

p value = 4.46 X 10^{-4} (all Normal vs PE)

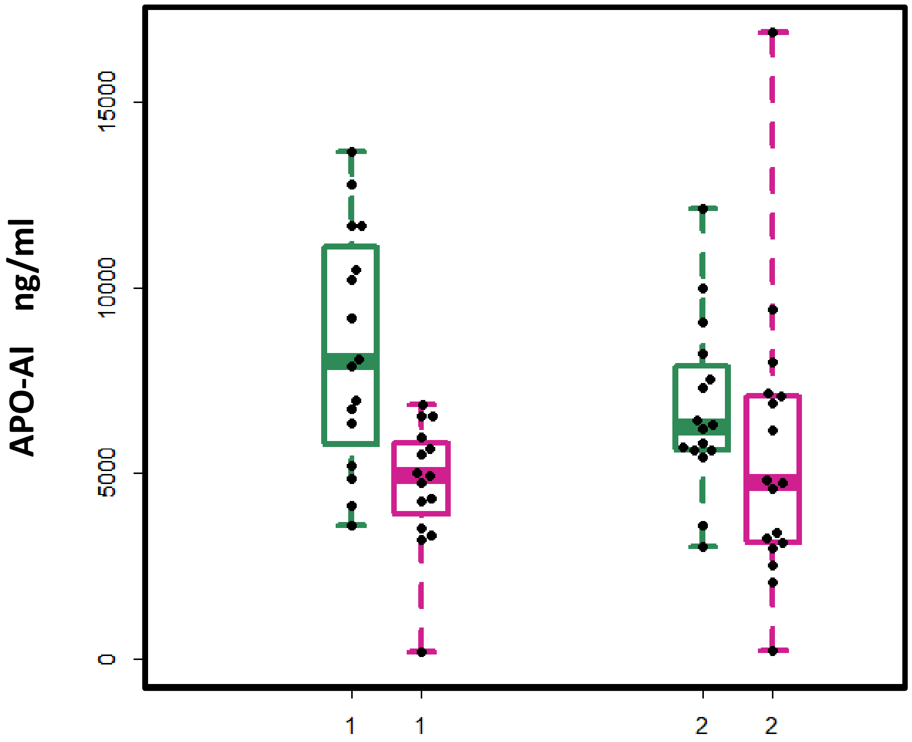

p value 1.67 X 10^{-3} 8.72 X 10^{-2}

GA 23-34 weeks		GA > 34 weeks	
Normal N=16	PE N=15	Normal N=16	PE N=17

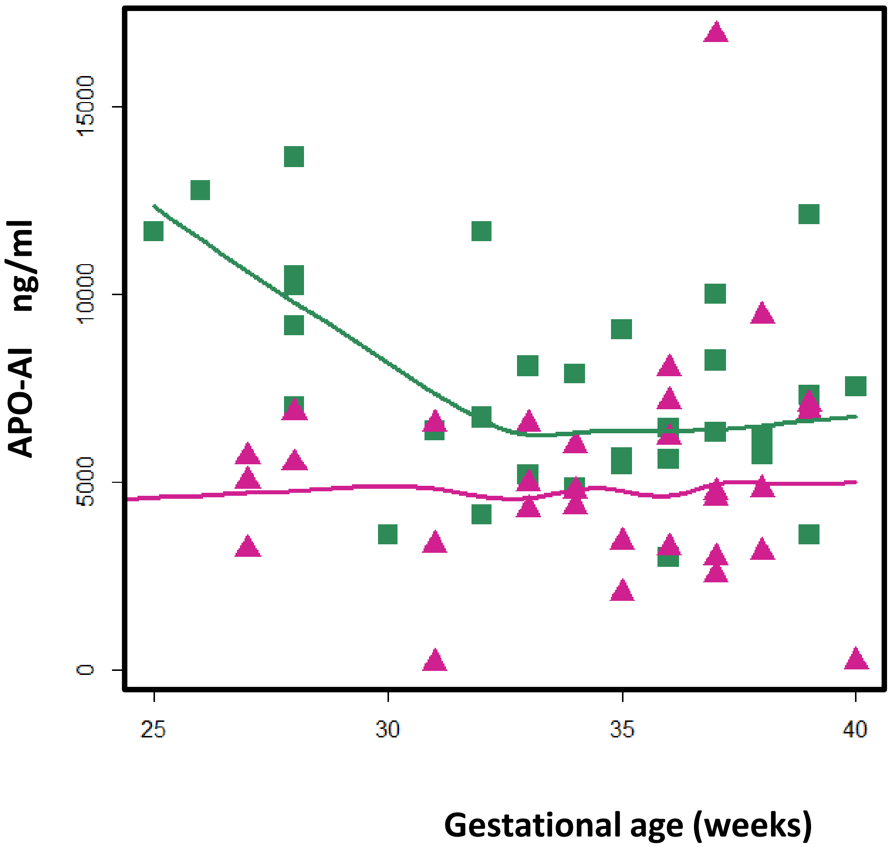

RBP4 Validation

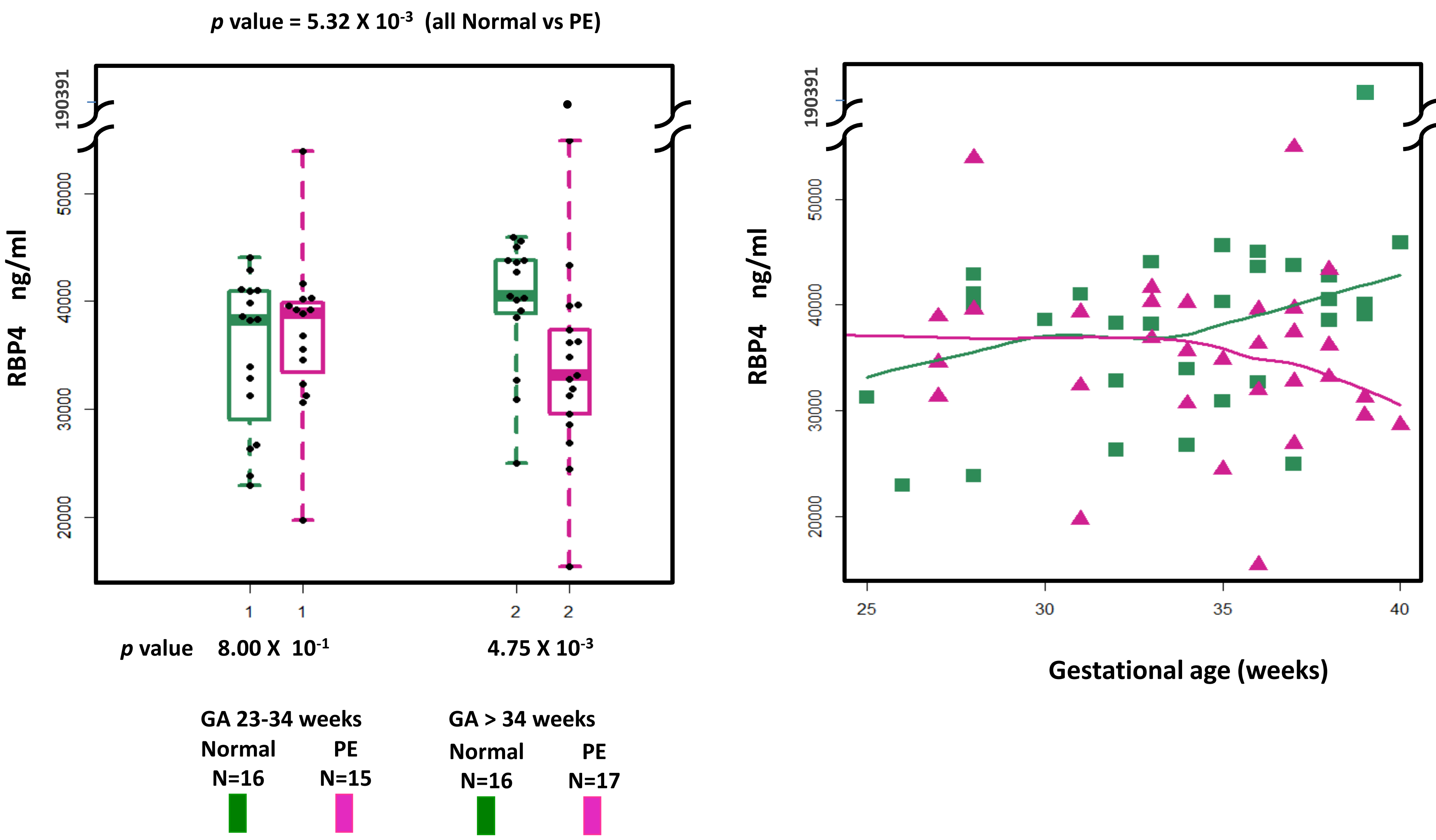

pikachurin Validation

p value = 1.92×10^{-8} (all Normal vs PE)

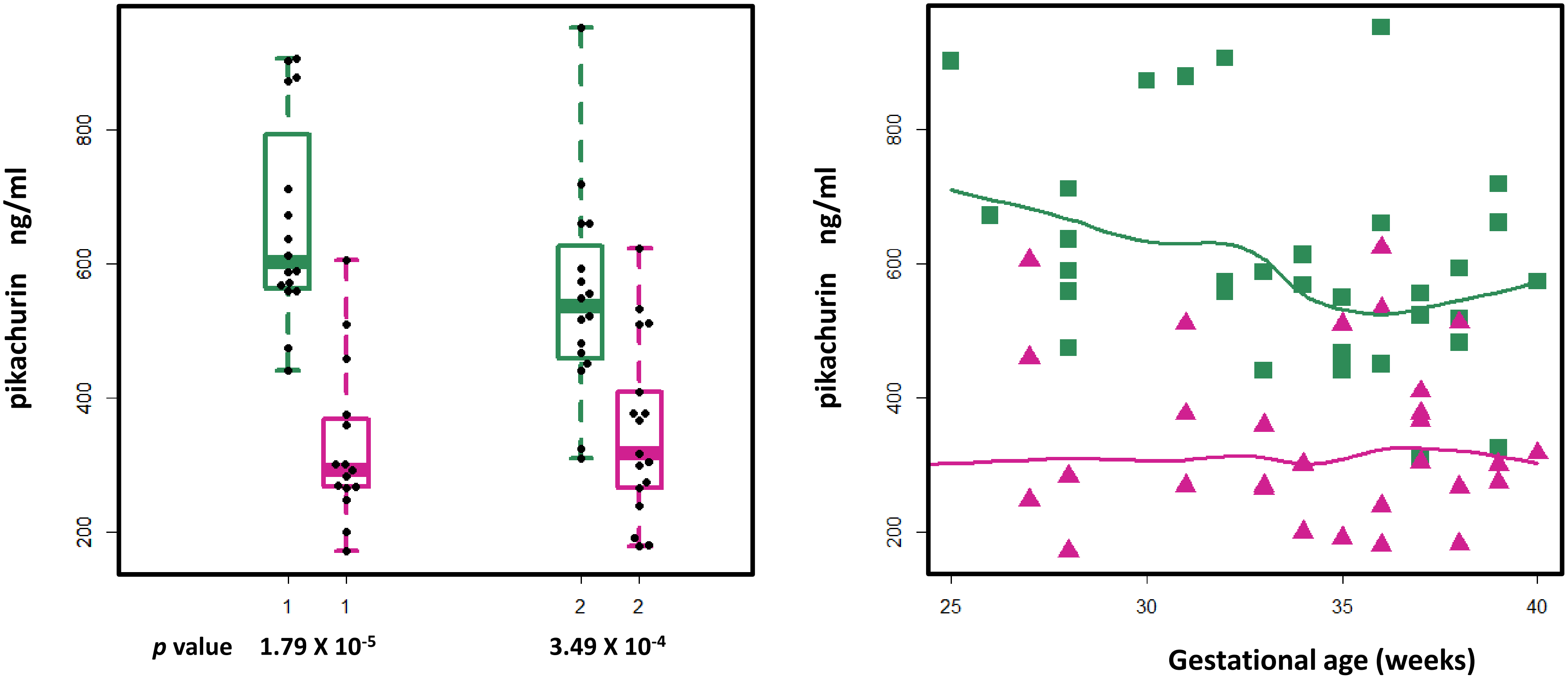

GA 23-34 weeks

Normal N=16 | PE N=15

GA > 34 weeks

Normal N=16 | PE N=17

Supplementary Figure 2

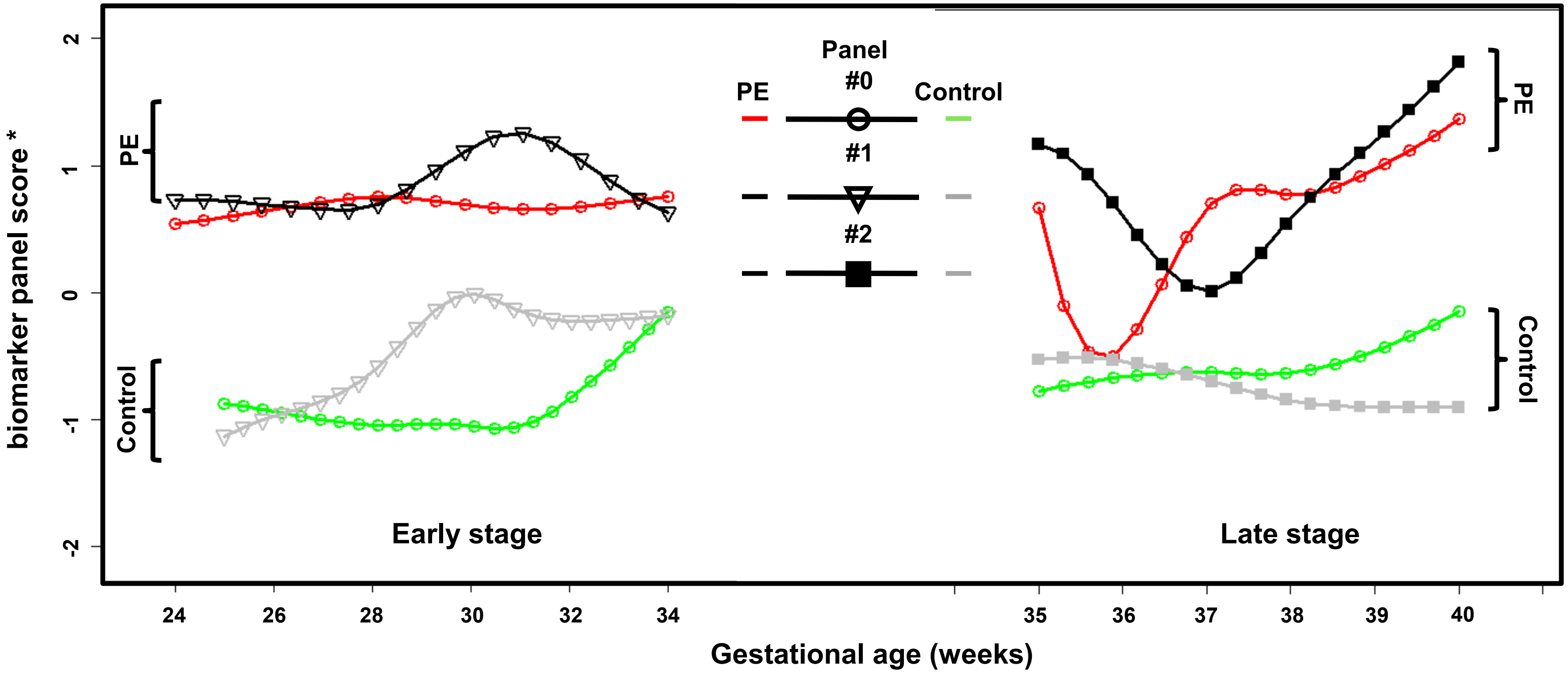

Supplementary Figure 3

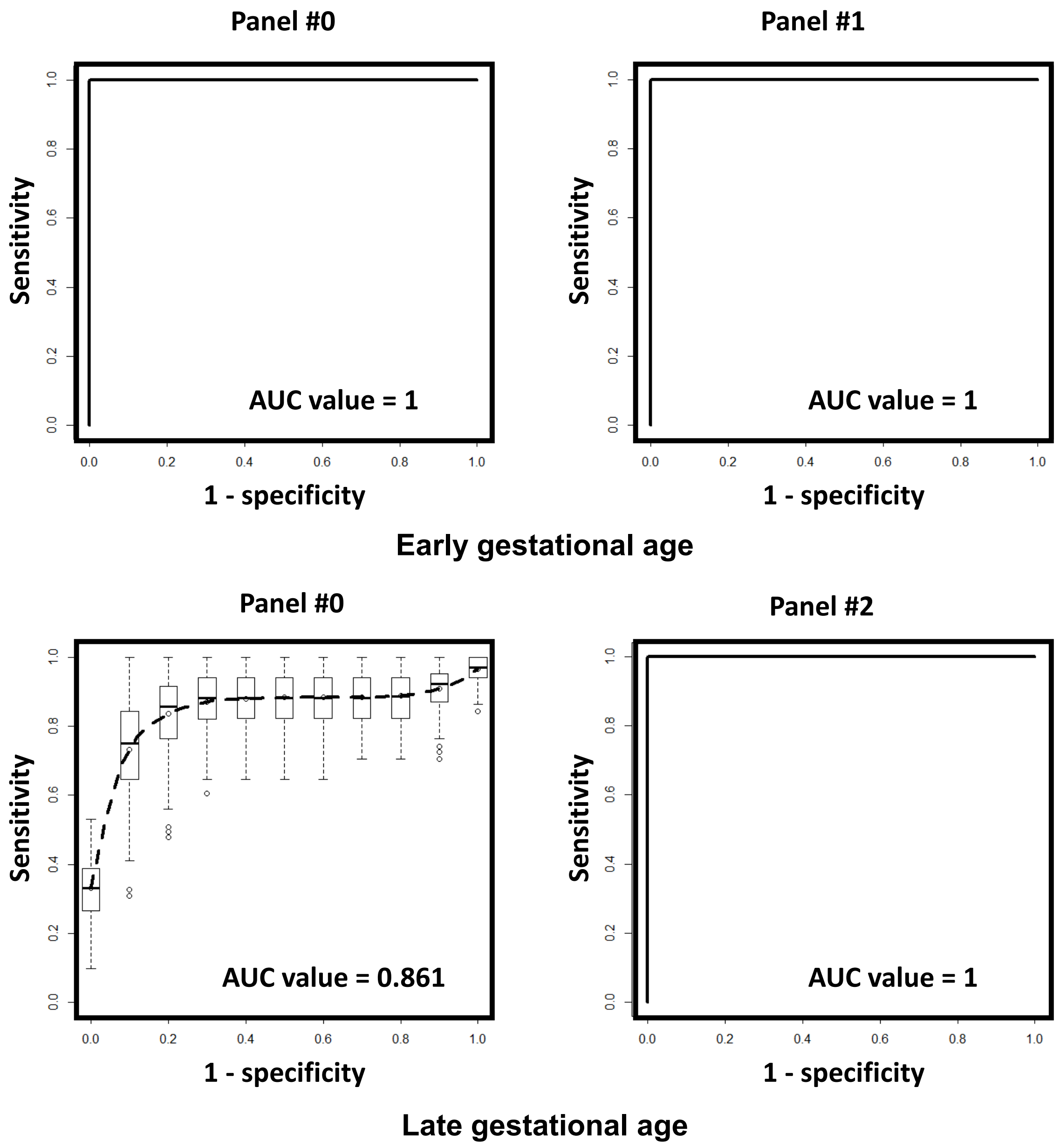

Supplement: Additional file 2: Figure S1 — Boxplot display and scatter plot of biomarker distributions at different gestation in PE and control groups. Horizontal box boundaries and midline denote sample quartiles. Figure S2. Composite overlay of different biomarker panels’ loess fitted lines for both PE and control subjects as a function of gestation. Figure S3. The performance, gauged by ROC analyses, of PE serum protein biomarker panel 0, 1, and 2 in discriminating PE and control subjects. [file 1741-7015-11-236-S2.pdf]
